# Maternal low-calorie sweetener consumption rewires hypothalamic melanocortin circuits via a gut microbial co-metabolite pathway

**DOI:** 10.1172/jci.insight.156397

**Published:** 2023-05-22

**Authors:** Soyoung Park, Amine M. Belfoul, Marialetizia Rastelli, Alice Jang, Magali Monnoye, Hosung Bae, Anna Kamitakahara, Patrick Giavalisco, Shan Sun, Pierre-Yves Barelle, Jasmine Plows, Cholsoon Jang, Anthony Fodor, Michael I. Goran, Sebastien G. Bouret

**Affiliations:** 1 The Saban Research Institute, Developmental Neuroscience Program, Children’s Hospital Los Angeles, University of Southern California, Los Angeles, California, USA.; 2 University Lille, Inserm, CHU Lille, Laboratory of development and plasticity of the Neuroendocrine brain, Lille Neuroscience & Cognition, Inserm UMR-S1172, Lille, France.; 3 Université Paris-Saclay, INRAE, AgroParisTech, Micalis Institute, Jouy-en-Josas, France.; 4 Department of Biological Chemistry, School of Medicine, University of California at Irvine, Irvine, California, USA.; 5 Metabolomics Core Facility, Max Planck Institute for Biology of Ageing, Cologne, Germany.; 6 Department of Bioinformatics and Genomics, College of Computing and Informatics, University of North Carolina Charlotte, Charlotte, North Carolina, USA.; 7 Center for Endocrinology, Diabetes and Metabolism, Children’s Hospital Los Angeles, University of Southern California, Los Angeles, California, USA.

**Keywords:** Metabolism, Neuroscience, Imprinting, Melanocortin, Neuroendocrine regulation

## Abstract

The prevalence of obesity and type 2 diabetes is growing at an alarming rate, including among pregnant women. Low-calorie sweeteners (LCSs) have increasingly been used as an alternative to sugar to deliver a sweet taste without the excessive caloric load. However, there is little evidence regarding their biological effects, particularly during development. Here, we used a mouse model of maternal LCS consumption to explore the impact of perinatal LCS exposure on the development of neural systems involved in metabolic regulation. We report that adult male, but not female, offspring from both aspartame- and rebaudioside A–exposed dams displayed increased adiposity and developed glucose intolerance. Moreover, maternal LCS consumption reorganized hypothalamic melanocortin circuits and disrupted parasympathetic innervation of pancreatic islets in male offspring. We then identified phenylacetylglycine (PAG) as a unique metabolite that was upregulated in the milk of LCS-fed dams and the serum of their pups. Furthermore, maternal PAG treatment recapitulated some of the key metabolic and neurodevelopmental abnormalities associated with maternal LCS consumption. Together, our data indicate that maternal LCS consumption has enduring consequences on the offspring’s metabolism and neural development and that these effects are likely to be mediated through the gut microbial co-metabolite PAG.

## Graphical abstract

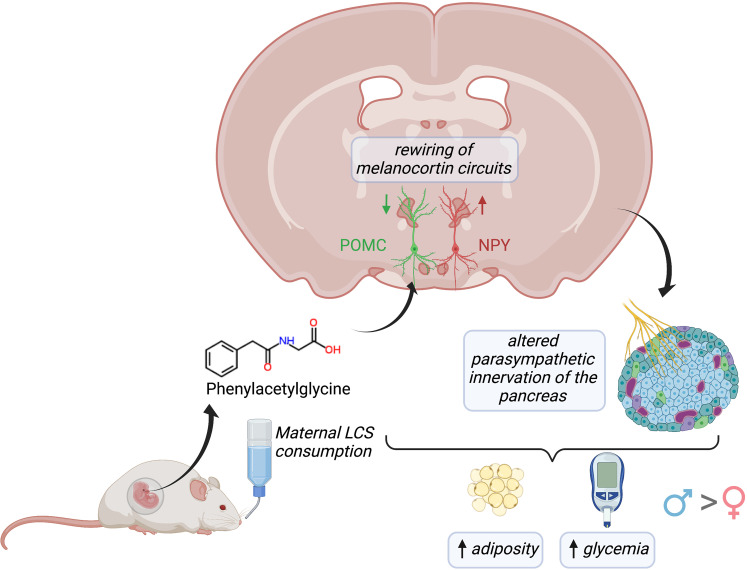

## Introduction

Dramatic changes in our nutritional environment have contributed to the current obesity epidemic. One of the most significant factors that has been linked to obesity is the marked increase in refined sugar consumption, particularly in the form of sugar-sweetened beverages (1, 2). In response, low-calorie sweeteners (LCSs) have gained immense popularity over recent decades as potentially healthier alternatives. LCSs seem ideal for weight maintenance as they retain the sweet flavor profile of sugar without the excess caloric intake. However, contrary to the intended benefits of LCSs, studies in rodents and humans suggest that LCS consumption is associated with weight gain and glucose intolerance (3–5). These conflicting results demand further studies because while LCSs are generally recognized as safe by the Food and Drug Administration and are heavily consumed, our knowledge on their biological actions in metabolically active systems remains limited.

In addition to their potential adverse effects on metabolism in adults, certain LCSs are found in the placenta, amniotic fluid, and breast milk (6–8). In addition, epidemiological evidence suggests that exposure to LCSs in utero may increase the risk of weight gain and obesity in children (9–12). However, these studies have all been observational and determining causation is challenging. Given the ethical barriers involved in randomized controlled trials assigning specific types of LCS exposure exclusively during pregnancy and/or lactation in humans, animal studies are necessary to ascertain if early-life exposure to certain LCSs affects the offspring’s metabolism. Existing studies in rodents suggest that in utero LCS exposure increases sweet taste preference in adults (13, 14). Moreover, maternal LCS consumption in rodents leads to elevated body weight and adiposity along with insulin resistance in the offspring and associates with changes in gut microbiota composition (11, 15, 16).

Because perinatal exposure to LCSs appears to cause long-term metabolic perturbations, it may alter the development of central pathways that are critical for energy metabolism regulation, in particular the hypothalamus. The hypothalamus is involved in the regulation of energy expenditure and glucose homeostasis (see ref. 17 for review). It is also the prime target of the developmental programming of obesity induced by maternal and perinatal nutritional imbalances (18–21). A critical component of the hypothalamic neuronal circuits is the melanocortin system consisting of the anorexigenic pro-opiomelanocortin (POMC) and the orexigenic agouti-related protein (AgRP) neurons in the arcuate nucleus (ARH). These neurons exert opposite and complementary effects on feeding and metabolism, particularly through their projections to the paraventricular nucleus of the hypothalamus (PVH) (17). In addition to hypothalamic circuits, the autonomic nervous system plays an important role in glucose homeostasis (22). Previous studies, including from our laboratory, have shown that maternal obesity disrupts hypothalamic development and alters the parasympathetic innervation of pancreatic β cells in the offspring (23, 24). However, whether nutritional insults other than a high-fat diet affect the development of hypothalamic melanocortin circuits or their downstream autonomic pathways remains unknown.

In the present study, we investigated whether consumption of commonly used LCSs — aspartame and rebaudioside A — affects the perinatal programming of metabolic disorders and the development of hypothalamic melanocortin and autonomic circuits. We found that maternal LCS exposure caused metabolic alterations, characterized by elevated fat mass, glucose intolerance, hyperleptinemia, and gut dysbiosis in the adult male offspring. We also reported that maternal LCS consumption remodeled hypothalamic melanocortin circuits and disrupted parasympathetic innervation of the pancreatic islets in the adult male offspring. Our study also identified phenylacetylglycine (PAG) as a metabolite accumulating with maternal LCS consumption. We further showed that maternal PAG treatment recapitulated many of the outcomes of maternal LCS consumption, including glucose intolerance, remodeling of hypothalamic melanocortin circuits, and alterations in parasympathetic innervation of pancreatic islets.

## Results

### Literature review on the impact of maternal LCS consumption on baby’s obesity risk.

To examine if maternal LCS consumption is associated with metabolic malprogramming in humans, PubMed, Ovid Medline, and Web of Science databases were systematically searched and identified 130 records. After removing duplicates, 54 records remained. Outcome measures were parameters of the offspring’s weight, BMI, and body composition. After excluding nonhuman studies, review articles, studies not in pregnancy/lactation, studies with incorrect outcomes, and duplicates, we identified 6 studies of interest to be included in the review (Supplemental Figure 1; supplemental material available online with this article; https://doi.org/10.1172/jci.insight.156397DS1) (9–12, 25, 26). A summary of these articles and their findings is shown in Supplemental Table 1. All identified studies were observational prospective cohort studies. A positive association between maternal LCS intake and offspring BMI *z* score, fat mass, and/or overweight and obesity was consistent across 5 out of 6 of these studies (9–12, 25, 26). Notably, 2 studies found that these effects were seen only in male infants when the analysis was stratified by sex (9, 10). Together, these clinical studies suggest that maternal LCS consumption is associated with metabolic malprogramming in humans. However, conclusions about causality could not be inferred from any of these studies given their observational nature.

### LCS consumption during pregnancy and lactation affects dams’ body composition.

**Figure 1:**
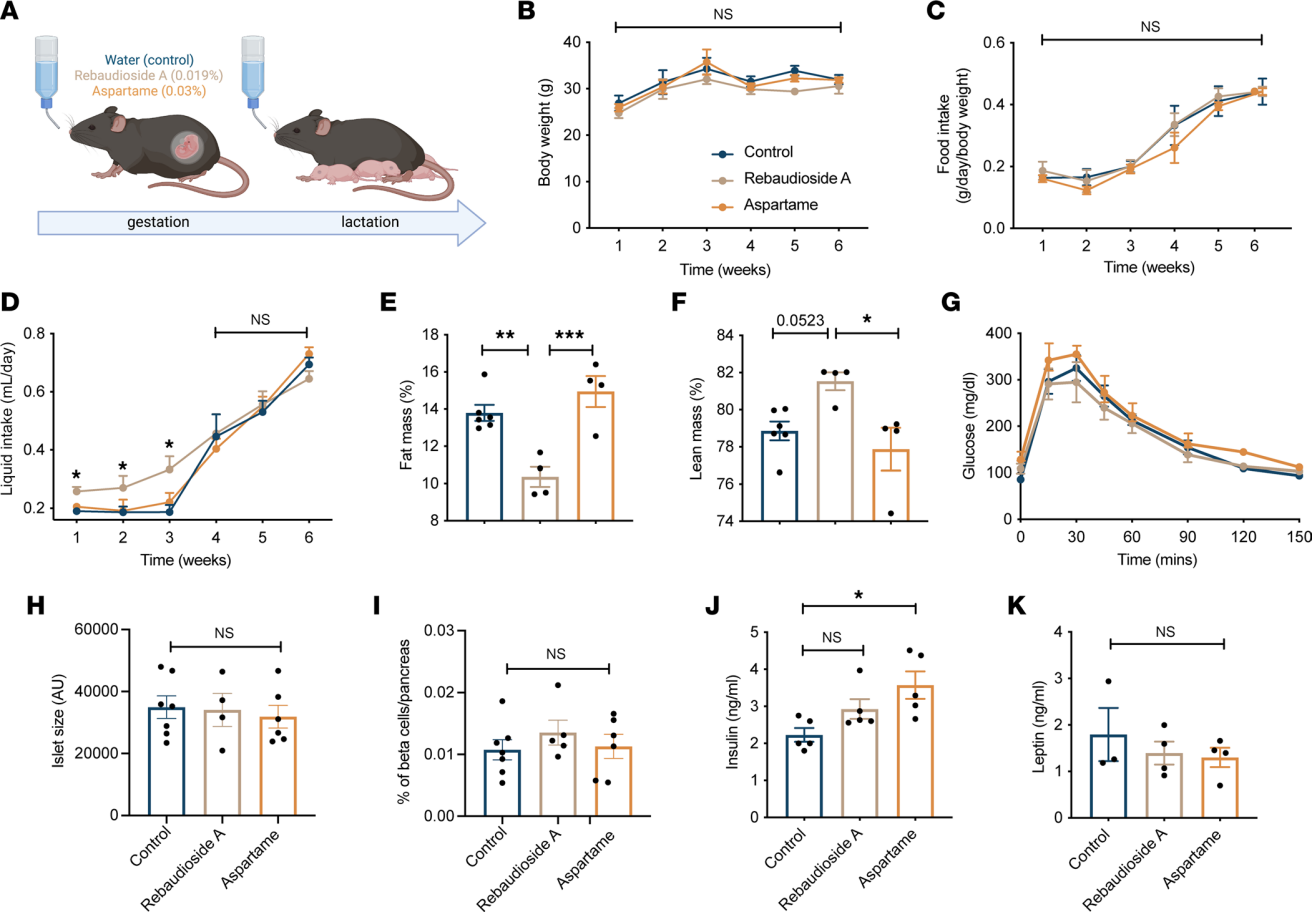
Impact of LCS consumption during pregnancy and lactation on the dam’s metabolism. (**A**) Experimental overview of the maternal LCS consumption mouse model. (**B**) Body weight curves, (**C**) average food intake, and (**D**) liquid intake in dams consuming a water (control), rebaudioside A, or aspartame solution during pregnancy and lactation (*n* = 5–7 dams per group). (**E**) Fat mass, (**F**) lean mass, and (**G**) glucose tolerance tests at the end of lactation in dams consuming a water (control), rebaudioside A, or aspartame solution (*n* = 4–6 dams per group). (**H**) Quantification of islet size and (**I**) percentage of β cell mass in the pancreas of dams consuming a water (control), rebaudioside A, or aspartame solution (*n* = 5–6 dams per group). (**J**) Serum insulin level at the end of lactation and (**K**) serum leptin level at P14 of dams consuming a water (control), rebaudioside A, or aspartame solution (*n* = 3–5 dams per group). Data are presented as mean ± SEM. Statistical significance between groups was determined by 2-way ANOVA followed by Bonferroni’s multiple-comparison test (**B**–**D** and **G**) or 1-way ANOVA followed by Tukey’s multiple-comparison test (**E**, **F**, and **H**–**K**). *P ≤ 0.05, **P ≤ 0.01, and ***P ≤ 0.001.

Because randomized controlled trials analyzing LCS consumption and effects during pregnancy in humans would not be ethical, rodent studies are needed to understand if there is indeed a causal relationship between maternal LCS intake and the offspring’s metabolic disturbances and to explore the biological mechanisms underlying these effects. We, therefore, developed an animal model in which adult C57BL/6J female mice were exposed to a chow diet combined with water (control), aspartame (0.03%), or rebaudioside A (0.02%) in the drinking bottle throughout gestation and lactation (Figure 1A). The doses of aspartame (equivalent of 2.4–7.0 mg/kg BW/d) and rebaudioside A (equivalent of 1.5–4.0 mg/kg BW/d) are well within the admissible daily intake in humans, which is 40 mg/kg BW/d for aspartame and 4 mg/kg BW/d for rebaudioside A. Dams’ body weight and food intake were not affected by LCS consumption (Figure 1, B and C). However, there was a significant increase in liquid intake in rebaudioside A–exposed dams during pregnancy, but liquid intake was normal during the lactation period (Figure 1D). Despite no differences in body weight, rebaudioside A–fed dams showed body composition redistribution with a reduction in fat mass and an increase in lean mass (Figure 1, E and F). LCS exposure did not affect glucose tolerance in dams (Figure 1G). Consistent with these data, islet size and percentage of β cell mass were normal in LCS dams (Figure 1, H and I). Serum insulin levels were increased in aspartame-fed dams (Figure 1J), but LCS exposure did not affect dams’ leptin levels (Figure 1K).

### Maternal LCS consumption increases fat mass and causes glucose impairment in the male offspring.

**Figure 2:**
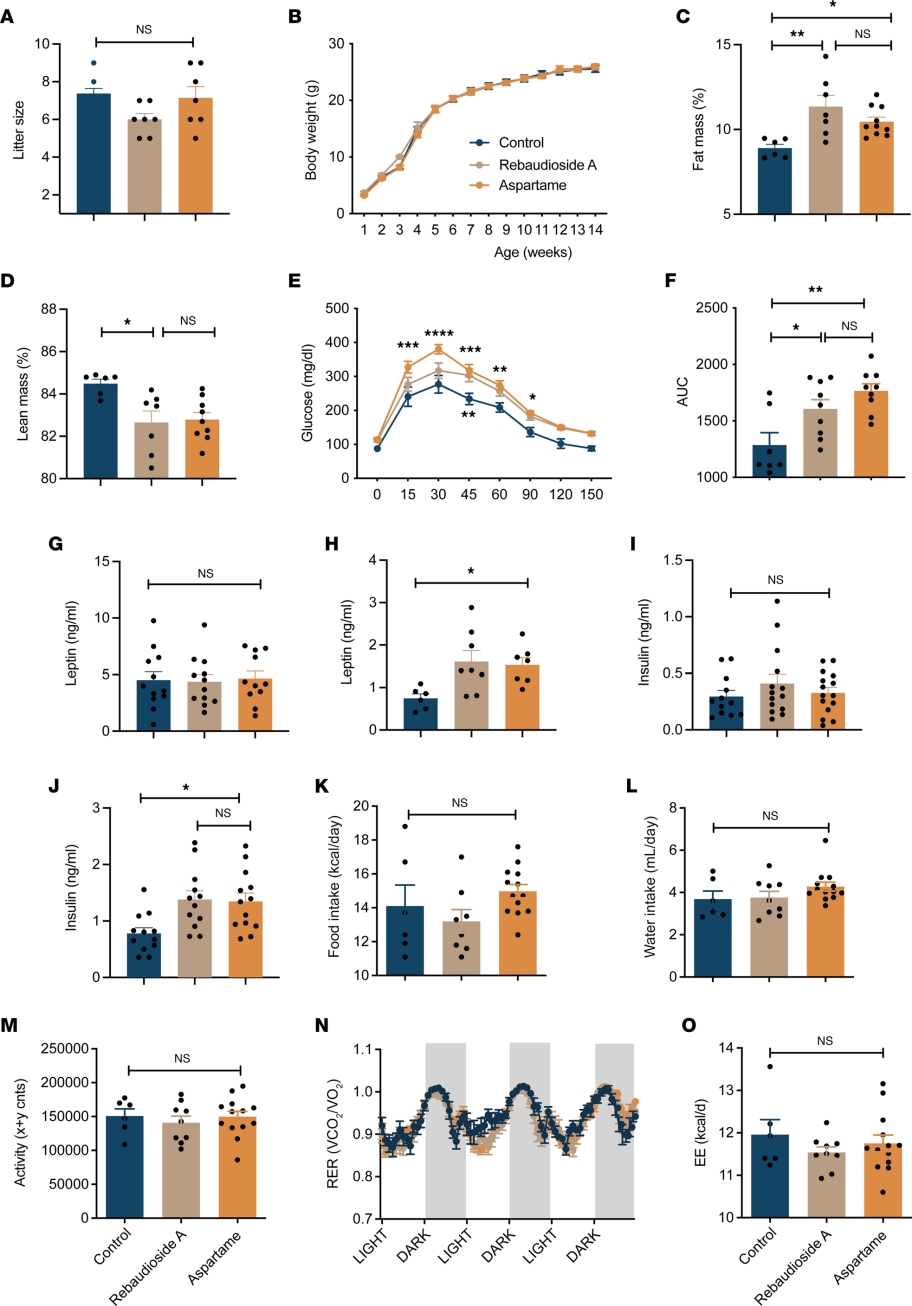
Maternal LCS consumption alters body composition and causes glucose intolerance in the male offspring. (**A**) Litter size of dams consuming a water (control), rebaudioside A, or aspartame solution (*n* = 4–7 dams per group). (**B**) Body weight curves of male mice born to dams consuming a water (control), rebaudioside A, or aspartame solution (*n* = 9–10 animals per group). (**C**) Fat and (**D**) lean mass of 14-week-old male mice born to dams consuming a water (control), rebaudioside A, or aspartame solution (*n* = 6–10 per group). (**E**) Glucose tolerance tests and (**F**) areas under the curve of 11- to 12-week-old male mice born to dams consuming a water (control), rebaudioside A, or aspartame solution (*n* = 7–9 animals per group). (**G** and **H**) Serum leptin and (**I** and **J**) insulin levels in (**G** and **I**) P14 and (**H** and **J**) 14-week-old male mice born to dams consuming a water (control), rebaudioside A, or aspartame solution (*n* = 6–15 animals per group). (**K**) Food intake, (**L**) water intake, (**M**) locomotive activity, (**N**) respiratory exchange ratio (RER), and (**O**) energy expenditure in 14-week-old male mice born to dams consuming a water (control), rebaudioside A, or aspartame solution (*n* = 6–13 animals per group). Data are presented as mean ± SEM. Statistical significance between groups was determined by 2-way ANOVA followed by Bonferroni’s multiple-comparison test (**B**, **E**, and **N**) or 1-way ANOVA followed by Tukey’s multiple-comparison test (**A**, **C**, **D**, **F**–**M**, and **O**). *P ≤ 0.05, **P ≤ 0.01, ***P ≤ 0.001, ****P ≤ 0.0001.

Litter size of both rebaudioside A– and aspartame-fed dams was normal at birth (Figure 2A). Mice born to either rebaudioside A– or aspartame-fed dams displayed normal pre- and postweaning growth curves compared to control mice (Figure 2B). However, adult male offspring of LCS-fed dams had elevated fat mass and lower lean mass compared with offspring of control dams (Figure 2, C and D). Moreover, maternal LCS consumption was associated with glucose intolerance in the adult male offspring (Figure 2, E and F). Maternal aspartame or rebaudioside A consumption did not affect pups’ leptin or insulin levels at P14 (Figure 2, G and I). However, the male adult offspring of rebaudioside A– and aspartame-fed dams displayed higher serum leptin and insulin levels (Figure 2, H and J). Food intake, water intake, locomotor activity, respiratory exchange ratio (RER), and energy expenditure were not changed in the adult male offspring of LCS-fed dams (Figure 2, K–O). The metabolic effects of perinatal LCS exposure appear sex specific because no significant changes in body weight, fat and lean mass, glucose tolerance, leptin and insulin levels, food intake, water intake, locomotor activity, RER, and energy expenditure were observed in female adult offspring born to either rebaudioside A– or aspartame-fed dams (Supplemental Figure 2, A–K).

### Maternal LCS consumption does not alter dams’ gut microbiota composition but affects the offspring’s microbiota signature during adult life.

It is now accepted that the maternal gut microbiota influences the offspring’s metabolism (27) and development (28). Based on these findings, we assessed the abundance and the composition of the fecal microbiota of dams consuming water, rebaudioside A, or aspartame during gestation and lactation. Fresh feces were collected at P14. The abundance of total bacteria, measured by quantitative PCR (qPCR) of 16S rDNA, was not affected by LCSs (Supplemental Figure 3A). We also examined dams’ fecal microbiota diversity and composition by using 16s rRNA gene sequencing followed by dada2 analysis of amplicon sequence variants (ASVs). Control and LCS-treated dams’ fecal microbiota did not significantly separate on the α-diversity (diversity within samples) (Supplemental Figure 3B) or β-diversity analysis (diversity between samples). In fact, in the principal component analysis plot, the fecal microbiota of LCS-consuming dams clustered together with that from control dams (Supplemental Figure 3C). Additionally, the profile of the fecal microbiota of LCS-treated dams did not differ from that of control dams, since the relative abundance of the most abundant bacterial phyla (Supplemental Figure 3D) and families (Supplemental Figure 3E) were both similar. Taken together, these data indicate that maternal LCS exposure does not markedly shift the abundance or the composition of maternal fecal microbiota during the lactating period.

We also assessed the gut microbiota diversity in the adult offspring of LCS-fed and control dams using 16S rRNA gene sequencing followed by dada2 analysis of fecal samples. We identified and classified 1,604 unique ASVs. In agreement with previous studies (29), we found that bacteria within the Bacteroidales family S24-7 and Lactobacillaceae family were dominant in the mouse gut microbiota (Supplemental Figure 4A). Principal component analyses of 16S rRNA gene sequencing data revealed a separation between the fecal microbiota of LCS-exposed mice and controls at PCoA4 (Supplemental Figure 4, B and C). Using linear mixed effects models, we also analyzed the influence of maternal LCS consumption on individual taxa. Maternal LCS exposure was associated with an increase in the abundance of Enterobacteriaceae family and a reduction of 3 ASVs belonging to S24-7 and *Escherichia shigella* (Supplemental Figure 3D).

Taken together, these data indicate that although LCS consumption during pregnancy and lactation does not substantially influence the abundance or the composition of the maternal fecal microbiota, it has a long-term effect on the offspring’s gut microbiota signature.

### Maternal LCS consumption rewires hypothalamic melanocortin circuits.

**Figure 3:**
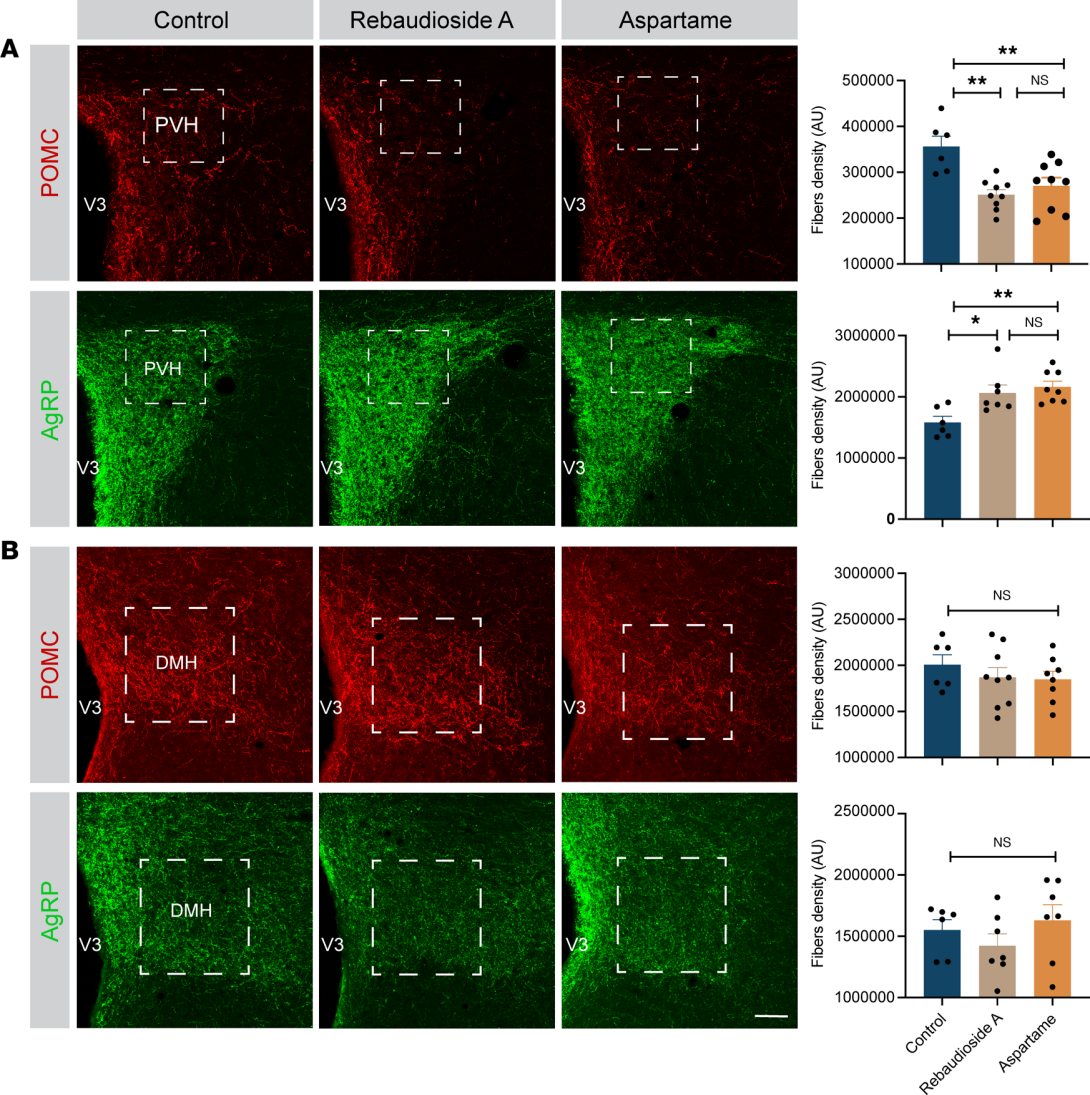
Maternal LCS consumption reorganizes hypothalamic melanocortin circuits. (**A** and **B**) Confocal images and quantification of the density of POMC- and AgRP-immunoreactive fibers innervating (**A**) the paraventricular nucleus of the hypothalamus (PVH) and (**B**) the dorsomedial nucleus (DMH) of 14-week-old male mice born to dams consuming a water (control), rebaudioside A, or aspartame solution (*n* = 6–9 animals per group). The white dotted area illustrates the quantified area. Data are presented as mean ± SEM. Statistical significance between groups was determined by 1-way ANOVA followed by Tukey’s multiple-comparison test. *P ≤ 0.05, and **P ≤ 0.01. Scale bars: 50 μm. V3, third ventricle.

The hypothalamic melanocortin system plays a critical role in regulating glucose and energy metabolism (30). Critical components of this system, including POMC and AgRP neurons, develop during midgestation and early postnatal life (31). To determine whether maternal LCS consumption affects the development of melanocortin circuits, we performed immunohistochemical labeling of POMC and AgRP in brain sections derived from adult mice born to LCS-fed or control dams. The density of POMC-immunoreactive fibers innervating the PVH was 1.4-fold lower in the adult offspring of both rebaudioside A– and aspartame-fed dams than that of mice born to control dams (Figure 3A). In contrast, the density of AgRP-labeled fibers innervating the PVH was 1.3-fold higher in adult male mice born to LCS-fed dams (Figure 3A). However, no significant changes in POMC and AgRP fiber densities were observed in female adult offspring born to either rebaudioside A– or aspartame-fed dams (Supplemental Figure 2, L and M). Moreover, the density of POMC- and AgRP-immunoreactive fibers innervating the dorsomedial nucleus of the hypothalamus (DMH) was normal in the male offspring of LCS-fed dams (Figure 3B). In addition, *Pomc* and *Npy* mRNA expression was not altered in male offspring of both rebaudioside A– and aspartame-fed dams (Supplemental Figure 5). Together, these data indicate that maternal LCS consumption causes a permanent rewiring of hypothalamic melanocortin circuits in the PVH.

### Maternal LCS consumption reduces parasympathetic innervation in the pancreas of male offspring.

**Figure 4:**
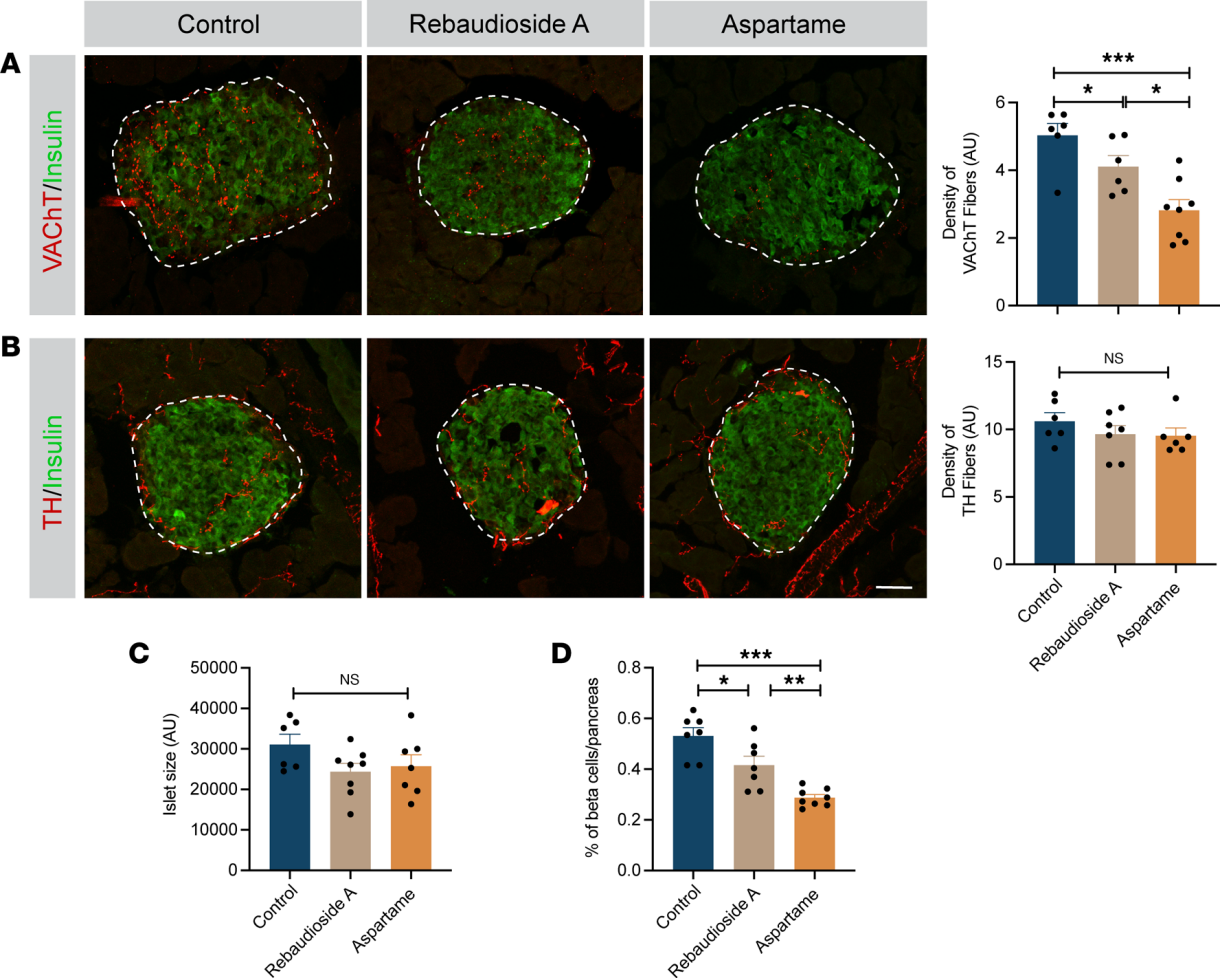
Maternal LCS consumption disrupts pancreatic parasympathetic innervation in the offspring. (**A** and **B**) Confocal images and quantifications of the density of (**A**) VAChT-immunoreactive (shown in red) and (**B**) TH-immunoreactive fibers (red) in insulin^+^ (green) islets of 14-week-old male mice born to dams consuming a water (control), rebaudioside A, or aspartame solution (*n* = 6–8 animals per group). The white dotted area illustrates the quantified area. (**C**) Quantification of islet size and (**D**) percentage of β cell mass in the pancreas of 14-week-old male mice born to dams consuming a water (control), rebaudioside A, or aspartame solution (*n* = 6–8 animals per group). Data are presented as mean ± SEM. Statistical significance between groups was determined by 1-way ANOVA followed by Tukey’s multiple-comparison test. *P ≤ 0.05, **P ≤ 0.01, and ***P ≤ 0.001. Scale bars: 50 μm.

The autonomic nervous system is a critical component of glucose homeostasis (22) and is a target of developmental programming (23, 32). Because mice born to LCS-exposed dams displayed glucose intolerance, we also analyzed the autonomic innervation of pancreatic islets by performing immunostaining for vesicular acetylcholine transporters (VAChTs) and tyrosine hydroxylase (TH), 2 surrogate markers for parasympathetic and sympathetic neurons, respectively. The density of VAChT-immunoreactive fibers innervating pancreatic β cells was 1.2- and 1.7-fold reduced in the male, but not the female, offspring of rebaudioside A– and aspartame-fed dams, respectively, compared with control mice (Figure 4A and Supplemental Figure 2N). However, the density of TH-labeled fibers in pancreatic islets appeared normal in the male and female offspring of LCS-exposed dams (Figure 4B and Supplemental Figure 2O). In addition, maternal LCS consumption did not affect pancreatic islet size (Figure 4C), but offspring from rebaudioside A– and aspartame-fed dams displayed reduced pancreatic β cell mass compared with offspring of control dams (Figure 4D). Together, these data suggest that maternal LCS consumption results in structural alterations of pancreatic islets in the offspring.

### Maternal LCS consumption changes the dam’s and offspring’s metabolomic profiling.

**Figure 5:**
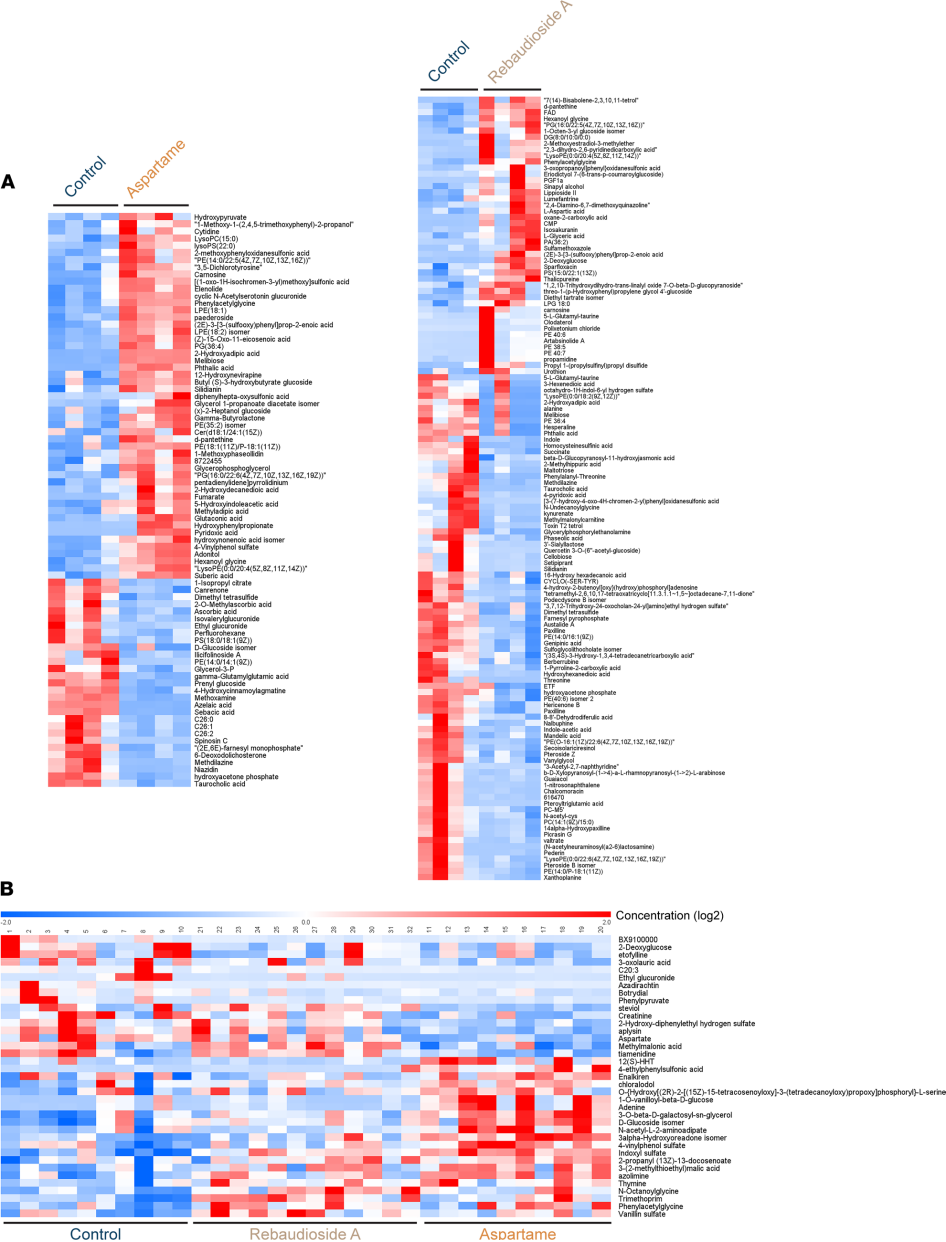
Maternal LCS consumption changes the metabolites in the adult offspring mice. (A and **B**) Heatmap of significantly enriched (*P* ≤ 0.05; FDR uncorrected) putative metabolites in (**A**) the breast milk of dams consuming a water (control), rebaudioside A, or aspartame solution (*n* = 4 animals per group) and (**B**) the serum of their pups at P10 (*n* = 7–8 animals per group).

We previously reported that aspartame and rebaudioside A do not affect axon growth from ARH explants ex vivo (33), suggesting that these LCSs affect melanocortin circuits indirectly. It is also known that after being ingested, LCSs are degraded into a variety of metabolites. Based on these observations, we performed an untargeted metabolomic analysis on milk and serum from P10 pups of rebaudioside A– and aspartame-fed dams. We identified 3,695 metabolomic features in maternal milk and 11,720 in pup sera. Maternal LCS consumption induced changes in metabolite profiles in both the dams’ milk and the pups’ sera (Figure 5).

**Figure 6:**
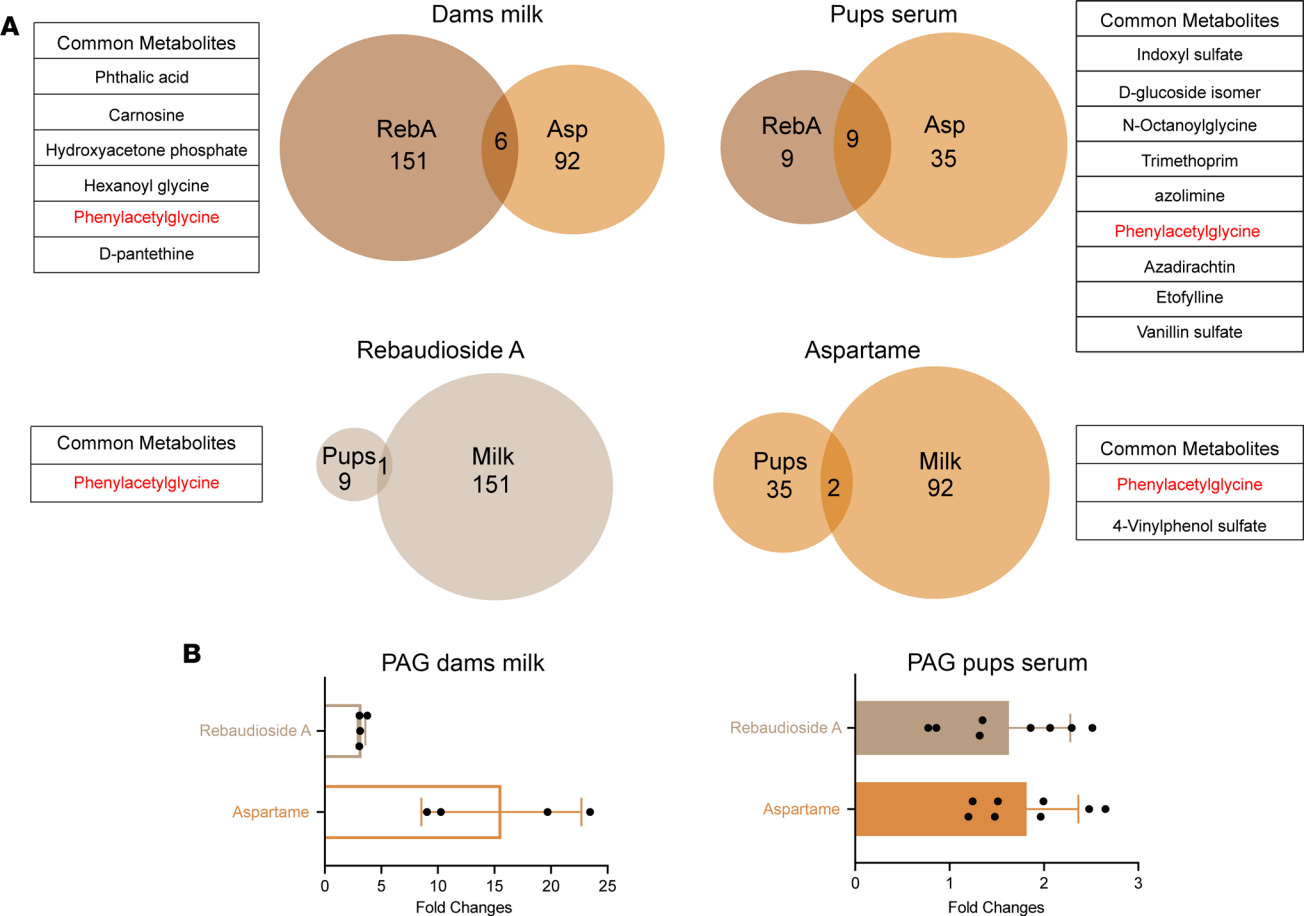
PAG is the only metabolite found in both the milk of LCS-fed dams and the serum of their pups. (**A**) Venn diagrams showing metabolites that are significantly enriched in the milk of dams consuming either a rebaudioside A (RebA) or an aspartame (Asp) solution and the serum of their pups at P10. (**B**) Fold-changes in PAG levels in the milk of dams consuming a water (control), rebaudioside A, or aspartame solution and the serum of their pups at P10 (*n* = 4 dams and *n* = 8 pups per groups). Data are presented as mean ± SEM.

A total of 151 annotated metabolites were differentially regulated (downregulated: 96 and upregulated: 55) in the milk from rebaudioside A–fed dams, and 92 metabolites were differentially regulated (downregulated: 31 and upregulated: 61) in the milk from aspartame-fed dams compared with controls (Figure 6A). We found 6 metabolites significantly changed in the milk from both aspartame-fed and rebaudioside A–fed dams (Figure 6A). In addition, a total of 9 metabolites were differentially regulated (downregulated: 2 and upregulated: 7) in the serum of P10 pups born to rebaudioside A–fed dams, and 35 metabolites were differentially regulated (downregulated: 15 and upregulated: 20) in the serum of pups born to aspartame-fed dams compared with controls (Figure 6A). Among them, 9 metabolites were similarly changed (downregulated: 2 and upregulated: 7) in the serum of P10 pups born to aspartame-fed and rebaudioside A–fed dams (Figure 6A). Remarkably, PAG, which is a gut microbe-host co-metabolite derived from phenylalanine catabolism and a biomarker of cardiovascular disease (34), was the only metabolite that was commonly changed (upregulated) in the milk of aspartame- and rebaudioside A–fed dams and the serum of their pups (Figure 6, A and B). These results indicate that maternal LCS consumption modulates biochemical profiles and that PAG may be a common metabolite mediating the programming effects of aspartame and rebaudioside A in the offspring.

### Maternal PAG exposure affects offspring’s glucose metabolism and autonomic innervation of the pancreas.

**Figure 7:**
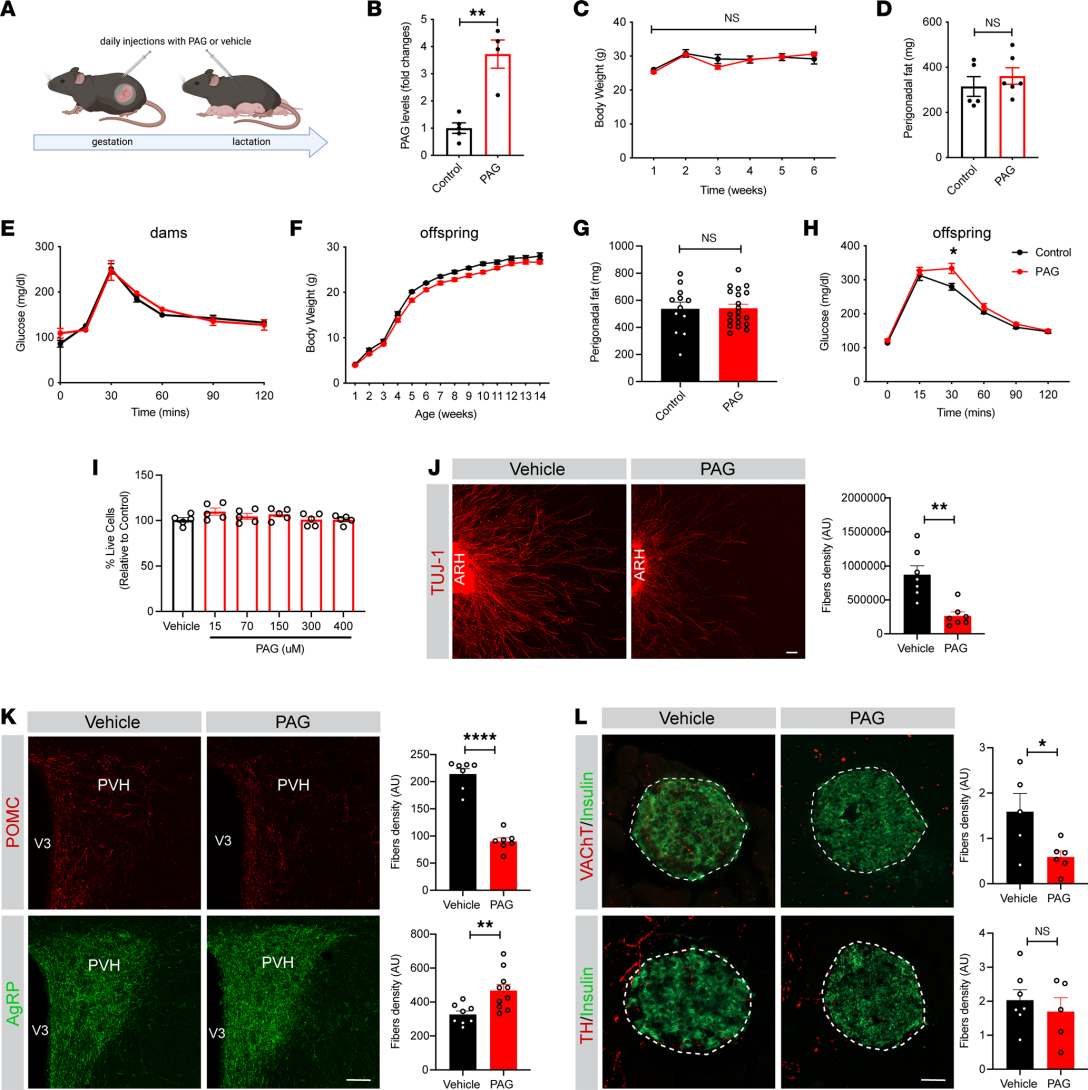
Maternal PAG treatment causes glucose intolerance and alters hypothalamic and autonomic circuits in the male offspring. (**A**) Experimental overview of the maternal PAG treatment. (**B**) Plasma PAG levels 30 minutes after intraperitoneal injection of 36.5 mg/kg of PAG in C57BL/6J female mice (*n* = 4–5 per group). (**C**) Body weight curves, (**D**) perigonadal fat mass, and (**E**) glucose tolerance tests of dams treated with PAG (36.5 mg/kg) or vehicle (control) (*n* = 5–6 dams per group). (**F**) Body weight curves, (**G**) perigonadal fat mass, and (**H**) glucose tolerance tests of 14-week-old male mice born to PAG- or vehicle-treated dams (*n* = 12–21 animals per group). (**I**) Percentage of live cells in hypothalamic mHypoE-N43/5 cells treated with saline or 15, 70, 150, 300, or 400 μM of PAG (*n* = 5 independent cultures per condition). (**J**) Confocal images and quantification of TUJ1^+^ (neuron-specific class III β-tubulin) fibers derived from isolated ARH explants incubated with vehicle or PAG (400 μM) (*n* = 7 independent cultures per condition). (**K**) Photomicroscopic images and quantification of the density of POMC and AgRP fibers innervating the PVH of 14-week-old male mice born to dams treated with PAG or vehicle (*n* = 7–10 animals per group). (**L**) Confocal images and quantifications of VAChT-immunoreactive (shown in red) and TH-immunoreactive fibers’ (red) density in insulin^+^ (green) islets of 14-week-old male mice born to PAG- or vehicle-treated dams (*n* = 4–7 animals per group). Data are presented as mean ± SEM. Statistical significance between groups was determined by 2-way ANOVA followed by Bonferroni’s multiple-comparison test (**C**, **E**, **F**, **H**, and **I**) or unpaired 2-tailed Student’s *t* test (**B**, **D**, **G**, and **J**–**L**). *P ≤ 0.05, **P ≤ 0.01, and ****P ≤ 0.0001. Scale bars: 50 μm.

To investigate the metabolic and neurodevelopmental effects of perinatal PAG exposure, we treated C57BL/6J female mice daily with PAG (36.5 mg/kg) or vehicle (saline) during pregnancy and lactation (Figure 7A). The dose of PAG that we used caused a 4-fold increase in plasma PAG levels in injected mice (Figure 7B). Dams’ body weight, perigonadal fat mass, and glucose tolerance were not affected by the PAG treatment (Figure 7, C–E). Mice born to PAG-treated dams displayed normal growth curves and fat mass compared to control mice (Figure 7, F and G). However, maternal PAG treatment was associated with glucose intolerance in the adult male offspring (Figure 7H). In contrast, maternal PAG exposure had no effect on growth curves, perigonadal fat mass, and glucose tolerance in female offspring (Supplemental Figure 2, P–R).

We next examined the effects of PAG on hypothalamic neurons. First, the mouse embryonic cell line N43/5 was exposed to a range of PAG concentrations (15, 70, 150, 300, 400 μM). We found that PAG did not affect hypothalamic cell viability (Figure 7I). We also exposed ARH explants from mouse neonates to PAG (400 μM) and evaluated its effects on axon growth ex vivo. After 48 hours, the density of axons from ARH explants treated with PAG was approximatively 2-fold lower than that of vehicle-treated explants (Figure 7J). In vivo, maternal PAG treatment induced a 2.4-fold decrease in the density of POMC-immunoreactive fibers in the PVH whereas it caused 1.4-fold increase in the density of AgRP-labeled fibers (Figure 7K). Moreover, the density of VAChT-immunoreactive fibers innervating pancreatic β cells was 2.7-fold reduced in the male offspring of PAG-treated dams (Figure 7L). However, the density of TH-labeled fibers in pancreatic islets appeared normal in the male offspring of PAG-exposed dams (Figure 7L).

Together, these data indicate that maternal PAG treatment recapitulates a variety of metabolic and neurodevelopmental abnormalities associated with maternal LCS consumption, suggesting that some of the programming effects of maternal LCSs are mediated, at least in part, through increased PAG levels.

## Discussion

A variety of studies have acknowledged that maternal obesity and diabetes alter the fetal and postnatal environments and contribute to adverse health outcomes for the developing offspring (35–37). One of the most significant factors that has been linked to obesity and type 2 diabetes is the marked increase in refined sugar consumption (1, 2). In response, LCSs have increasingly been used as an alternative to sugar in the attempt to deliver a sweet taste without the excessive caloric load. However, the effects of maternal LCS consumption on the offspring’s brain development and metabolic regulations are not fully understood. In this study, we show that maternal aspartame and rebaudioside A consumption during pregnancy and lactation causes lifelong metabolic alterations in the offspring characterized by increased fat mass, glucose dysregulations, and gut dysbiosis. We also report that maternal LCS consumption permanently rewires hypothalamic melanocortin circuits and disrupts the parasympathetic innervation of pancreatic β cells. Furthermore, our metabolomic analysis and maternal injection experiment revealed that the effects of LCSs on the offspring are likely to be indirect and could be mediated through the gut microbial co-metabolite PAG.

Reported LCS consumption has consistently been increased from 8.7% to 25.1% in children, and from 26.9% to 41.4% in adults from 1999–2000 to 2009–2012, with most consumers reporting consumption at least once a day (80% of children and 56% of adults). LCS consumption has also been reported to be higher in women than men (38). Data from the Danish National Birth Cohort indicated that almost half of women with gestational diabetes consumed beverages with LCSs during pregnancy, with 9.3% consuming them daily (10). Although initially LCSs were thought to be safe, an increasing number of research studies have suggested that LCS are not inert compounds and can exert long-term health and metabolic effects. For example, our literature review revealed that 5 human studies found a positive association between maternal LCS intake and offspring BMI *z* score, fat mass, and/or overweight and obesity (9–12, 25, 26). Our findings in mice are generally consistent with previous studies in rodents that have demonstrated that maternal LCS consumption in mice leads to increased fat mass in the adult male offspring (11). Notably, we found that the effects of maternal LCS consumption are sex specific, with female offspring not being affected. These data are consistent with 2 human studies reporting that the impact of LCS consumption in infant overweight was only apparent in male, but not female, infants (9, 10). Because overweight adults and adults with obesity drink more diet beverages than healthy-weight adults (39), and because maternal obesity predisposes offspring to metabolic diseases and disrupts hypothalamic development (23, 24, 40, 41), it would also be relevant to study whether similar programming effects of maternal LCS consumption are observed in an obesogenic environment. A recent study found that maternal aspartame and stevia consumption coupled with a high-fat/high-sucrose diet results, similar to our study in chow-fed dams, in elevated body weight, insulin insensitivity, and gut dysbiosis in the adult offspring (15). Together, these observations indicate that maternal LCS consumption disrupts metabolic regulations independently of maternal diet, with a greater programming effect in the male offspring.

After ingestion, LCSs bind to sweet taste receptors to induce their sweet effects. Notably, sweet taste receptors are found not only in the tongue but also in various parts of the body, including the CNS. Although certain LCSs (e.g., sucralose, acesulfame K, and saccharin) circulate in the body unmetabolized and are found in the blood, urine, amniotic fluid, cord blood, and breast milk, others (such as aspartame and rebaudioside A) are fully degraded in the gastrointestinal tract (42, 43). Based on these observations, it is not surprising that our metabolomic analysis did not detect aspartame or rebaudioside A in the maternal milk or neonate blood. Therefore, the effects of maternal aspartame and rebaudioside A consumption on the offspring’s hypothalamic development and metabolism are likely to be indirect. Consistent with this idea, we previously reported that direct exposure of hypothalamic arcuate explants ex vivo to these LCSs does not affect axon growth (33). Nevertheless, maternal aspartame and rebaudioside A exert very similar effects on the offspring’s metabolism and hypothalamic development, suggesting that both LCSs, although structurally different, may mediate their neuro-programming effects through a common mechanism. Our metabolomic analysis identified metabolites that were specifically enriched in the milk of LCS-fed dams or serum of their pups. Among those enriched metabolites, we found 1 metabolite, PAG, that was commonly upregulated in the milk of both aspartame- and rebaudioside A–fed dams and the serum of their pups. These data suggest that the effects of maternal aspartame or rebaudioside A consumption in the offspring could be mediated, at least in part, through their metabolization into PAG by the microbiota and liver axis. Supporting this idea, maternal PAG treatment mimics key neurodevelopmental outcomes associated with maternal LCS consumption, including reorganization of hypothalamic melanocortin circuits, and disruption of parasympathetic innervation of pancreatic islets. Moreover, PAG treatment impaired glucose homeostasis. However, in contrast to LCS exposure, PAG treatment had no effects on fat mass. This latter finding suggests that other metabolites might mediate the effects of LCSs on adiposity. Future studies will also be needed to investigate whether PAG level is increased upon consumption of other LCSs that are known to induce metabolic malprogramming in the offspring, such as acesulfame K (AceK) and sucralose (11). However, because these LCSs can be found in the urine of neonates upon maternal consumption, it suggests that, in contrast to aspartame and rebaudioside A, sucralose and AceK could have direct effects on the developing neonate.

PAG is a gut microbial co-metabolite derived from phenylalanine (34). Following ingestion of phenylalanine, one of the main direct breakdown products of aspartame, unabsorbed phenylalanine that reaches the large intestine can be metabolized by gut microbiota to form phenylpyruvic acid and subsequently phenylacetic acid. After absorption into the portal system, phenylacetic acid is readily metabolized in the liver to produce PAG. An association between PAG and obesity, diabetes, and cardiovascular diseases has previously been reported (34, 44–46). A recent study showed that PAG levels decreased in the mouse brain from P3 to P21 (47), suggesting that it may also play a role in brain development. Consistent with this hypothesis, our ex vivo experiments revealed that PAG alters the overall axonal outgrowth from arcuate nucleus explants. It was not technically feasible to specifically label POMC and AgRP fibers in vitro because of the rapid release of these peptides in ex vivo conditions. However, we showed that when administrated in vivo, PAG has opposite effects on POMC and AgRP neural projections: it decreases the density of POMC circuits while it increases the density of AgRP neural pathways. The exact mechanisms by which PAG exerts its effects on hypothalamic development remain to be determined. However, PAG has been shown to transmit cellular responses via its interactions with GPCRs, including adrenergic receptors (34). Interestingly, a previous study reported that activation of adrenergic receptors by noradrenaline modulates the activity of NPY/AgRP and POMC neurons in opposite ways: it increases the activity of NPY/AgRP neurons and decreases the activity of POMC neurons (48). These findings could explain why aspartame and rebaudioside A exert an opposite effect on the development of POMC and AgRP circuits. Alternatively, it is possible that the increased density of AgRP fibers could negatively regulate POMC neuronal development since it is known that AgRP can have direct inhibitory effects on POMC neurons (49).

In addition to contributing to the increased levels of PAG, the gut microbiota could also be involved in the metabolic defects observed in animals perinatally exposed to LCSs. Dysfunction of gut microbiota has been associated with obesity and insulin resistance (see ref. 50 for review). The gut microbiota can be shaped by various factors, including diet, antibiotics, stress, and exercise (51). Maternal consumption of artificially sweetened beverages during pregnancy has been shown to influence the establishment of the gut microbiome in infants (12). The present data are consistent with a previous rodent study reporting that consumption of aspartame or rebaudioside A has a limited effect on the dam’s microbiota diversity and composition. However, we and Wang et al. (52) show that maternal LCS intake has more long-term effects on the offspring’s microbiome. In particular, we found an increase in Enterobacteriaceae and the ASV identified as *Escherichia shigella* in the adult offspring of LCS-fed dams. Similar to our findings, adult rats exposed to aspartame displayed an increased proportion of Enterobacteriaceae (53). Members of the Proteobacteria phylum, which includes Enterobacteriaceae, produce gases and short-chain fatty acids that have been previously linked with inflammation and insulin resistance (54).

In conclusion, the present study unraveled broad consequences of maternal LCS consumption on critical components of metabolic systems. It also provided potential mechanistic insights into how perinatal LCSs exert their effects on hypothalamic development by identifying PAG as a potential mediator of LCS on neurodevelopmental and metabolic outcomes.

## Methods

### Literature searches, search strategies, and eligibility criteria.

Systematic literature searches using the following prespecified terms were performed on PubMed, Ovid Medline, and Web of Science: “non-nutritive sweetener*”OR “artificial sweetener*” OR “low-calorie sweetener*” OR “alternative sweetener*” OR “diet soda” OR “diet beverage*” OR “sugar-free” OR “sugar free” OR “aspartame” OR “acesulfame-K” OR “acesulfame potassium” OR “stevia” OR “rebaudioside” OR “sucralose” AND “pregnan*” OR “gestat*” AND “obes*” OR “body mass index” OR “BMI” OR “weight.” No filters were applied to any of the searches. No language or location restrictions were applied. We screened the title and abstract of the resulting records to identify those that 1) were in humans, 2) were not review articles, 3) were in the population of interest (pregnancy/gestation), and 4) had the outcome of interest (offspring BMI *z* score, fat mass, or overweight/obesity risk).

### Animals and treatments.

C57BL/6J wild-type (WT) mice (The Jackson Laboratory and Janvier Labs) were used in this study. The animals were housed under specific pathogen–free conditions and maintained in a temperature-controlled room with a 12-hour light/ 12-hour dark cycle. At 7 weeks of age, mice were mated (*n* = 5–7 dams per group), and pregnancy was determined by the presence of a vaginal plug the next day. The day of conception (sperm-positive vaginal smear) was designated as E0.5. Pregnant mice were given ad libitum access to a chow diet and water (control), rebaudioside A (0.02%, MilliporeSigma catalog 01432), or aspartame (0.03%, MilliporeSigma catalog PHR1381) in a drinking bottle from conception until the end of the lactation period. Male breeders were fed a normal chow diet and drank water. Offspring were fed a normal chow diet and drank water after weaning. Litter sizes were standardized to 6 pups 48 hours postdelivery, and attempts were made to maintain an equal sex ratio.

For the maternal injections of PAG, C57BL/6J WT female mice (Janvier Labs) were treated daily with i.p. injections of PAG (36.5 mg/kg, MilliporeSigma catalog 96408) or vehicle (saline) starting on the mating day and until the end of lactation (*n* = 5–6 per group).

### Physiological measures.

Maternal body weight, food intake, and liquid intake were recorded weekly from the day of mating until the end of lactation. Offspring were weighed weekly from 1 to 14 weeks of age using an analytical balance. Body composition analysis (fat/lean mass) was performed on dams at the end of the lactation period and offspring at 12–13 weeks of age using NMR (Echo MRI). Food intake, O_2_ and CO_2_ production, energy expenditure, RER (i.e., VCO_2_/O_2_), and locomotive activity (XY) were monitored in offspring at 15–16 weeks of age using a combined indirect calorimetry system (TSE Systems). The mice were acclimated in monitoring chambers for 2 days, and the data were collected for 3 days. These physiological measures were performed at the Rodent Metabolic Core of Children’s Hospital of Los Angeles.

Glucose tolerance tests (GTTs) were conducted in dams at the end of lactation and offspring at 10–11 weeks of age through i.p. injection of glucose (1.5 mg/g body weight) after overnight fasting. Blood glucose levels were measured at 0, 15, 30, 45, 60, 90, 120, and 150 minutes postinjection, as previously described.

Serum leptin and insulin levels were assayed in dams and pups using commercially available leptin (MilliporeSigma, catalog EZML-82K) and insulin (Mercodia, catalog 10-1249-01) ELISA kits.

### Milk collection.

The dam was separated for approximately 14 hours prior to milking. Milk was collected when pups were aged P12 to P13. The dam was transported to the lab and anesthetized using isoflurane. Then, the milking procedure took place under the fume hood, where warm saline was applied to the nipples before milking, and a 1 mL syringe was used to grasp the nipple to make a suckling movement. A 20 μL pipet was used to collect the milk, and the sample was frozen immediately.

### Immunohistochemistry.

Thirteen- to 14-week-old mice were perfused transcardially with 4% paraformaldehyde. Brains were then frozen, sectioned at 30 μm thickness, and processed for immunofluorescence using standard procedures (24). The primary antibodies used for IHC were as follows: rabbit anti-POMC (1:20,000, Phoenix Pharmaceuticals catalog H-029-30) and rabbit anti-AgRP (1:1,000, Phoenix Pharmaceuticals catalog H-003-53). The primary antibodies were visualized with goat anti-rabbit Alexa Fluor 488 (1:200, Thermo Fisher Scientific catalog A11008) or anti-rabbit IgG Alexa Fluor 568 (1:200, Thermo Fisher Scientific catalog A11011) secondary antibodies.

Pancreatic tissues were dissected from perfused animals, frozen, cut at 20 μm thickness, and processed for immunofluorescence using standard procedures (24). The primary antibodies used for IHC were as follows: guinea pig anti-insulin (1:500, Genetex catalog GTX39371), rabbit anti-VAChT (1:500, Synaptic Systems catalog 139 103), and rabbit anti-TH (1:500, MilliporeSigma catalog Ab152). The primary antibodies were visualized with donkey anti–guinea pig Alexa Fluor 488 (1:200, Thermo Fisher Scientific catalog A11073) or donkey anti-rabbit Alexa Fluor 568 (1:200, Thermo Fisher Scientific catalog A10042) secondary antibodies.

All sections were counterstained with bis-benzamide (1:10,000, Invitrogen) to visualize cell nuclei.

### Image analysis.

The images were acquired through the PVH and the DMH and pancreas using a ZEISS LSM 710 confocal system equipped with a 20× objective. For the quantitative analysis of β cell mass, images were acquired using a 5× objective. Quantifications were performed in 2–4 sections per animal. Slides were numerically coded to obscure the treatment group. The image analysis was performed using ImageJ analysis software (NIH) as previously described (24).

For the quantitative analysis of fiber density (for POMC, AgRP, VAChT, and TH fibers), each image plane was binarized to isolate labeled materials from the background and compensate for differences in fluorescence intensity. The integrated intensity, which reflects the total number of pixels in the binarized image, was then calculated for each image as previously described (24). This procedure was conducted for each image plane in the stack, and the values for all the image planes in a stack were summed.

Mass of β cells was measured as previously described (24). Briefly, immunolabeled pancreatic sections (2 sections per animal) separated by at least 60 μm were acquired using a ZEISS LSM 700 confocal system equipped with a 5× objective. The β cell area and the total area of the pancreas were determined using ImageJ. The total pancreas area was calculated from *n* = 7–8 for each group.

### RNA extraction and quantitative real-time PCR analyses.

The ARH of P10 mice was dissected under a stereomicroscope (Leica Biosystems). Total RNA was then isolated using the Arcturus PicoPure RNA Isolation Kit (for hypothalamic samples) (Thermo Fisher Scientific). cDNA was generated with the High-Capacity cDNA Reverse Transcription Kit (Life Technologies). Quantitative real-time PCR was performed using TaqMan Fast Universal PCR Master Mix (Thermo Fisher Scientific, catalog 4352042) and the commercially available TaqMan gene expression primers: *Pomc* (Mm01319226_m1), *Npy* (Mm00445771_m1), and *Gapdh* (Mm99999915_g1). mRNA expression was calculated using the 2-ΔΔCt method after normalization to the expression of the *Gapdh* housekeeping gene. All assays were performed using an Applied Biosystems 7900 HT real-time PCR system.

### Microbiome analysis.

Dams’ fresh fecal samples were collected at P14 and in the offspring at 13–14 weeks of age. Samples were stored at −80°C until analysis. Total DNA was extracted using DNA extraction QIAamp PowerFecal Pro Kit (QIAGEN, catalog 51804). The V3-V4 hypervariable region of the 16S rRNA gene (55) was amplified using Phanta Max Super-Fidelity DNA Polymerase (Vazyme, catalog P505), and the primers forward MS341F: 5′-CCTACGGGNGGCWGCAG-3′ and reverse MS785R: 5′-GACTACHVGGGTATCTAATCC-3′ were used. They were selected from the Klindworth et al. publication (56). Quality control, sequencing library construction, and sequencing (MiSeq Illumina) were performed using the Genotoul bioinformatics platform. The resulting sequences were analyzed using R workflow combining dada2 v.1.26 (57) and FROGS 4.0.0 (58) software. Reads were filtered, merged, and assigned to ASVs with dada2, and the ASVs were assigned to species using the FROGS affiliation tool. Adapters were first removed using cutadapt v. 3.5. Reads were then filtered using the dada2 filterAndTrim function, with a truncation length of 200 bp for 16S forward and reverse reads. This truncation reduced the error rate while allowing the merging of most reads. The error model was then calculated using the learnErrors function. Then, the dada2 core sample inference algorithm was executed. Forward and reverse reads were then merged with a minimum overlap of 20 bp. The resulting sequences were saved in a sequence table using makeSequenceTable. The guidelines of FROGS 4.0.0 were followed to detect and remove chimeras using the vsearch tool. Operational taxonomic units with global abundance lower than 0.005% were removed from the following analysis with FROGS filters, and the ASVs in the sequence table were then assigned to species using FROGS affiliation with silva 138 (59).

16S rRNA amplicon sequencing bioinformatic analysis was performed on R (v4.2.1) with package phyloseq (v1.40.0). Differential analyses were performed with DESeq2 R package (v1.36.0).

To quantify the abundance of total bacteria, qPCR of 16S rDNA was performed using the primers F_Bact 1369: 5′-CGGTGAATACGTTCCCGG-3′ and R_Prok1492: 5′-TACGGCTACCTTGTTACGACTT-3′. PCR amplification was carried out as follows: 10 minutes at 95°C, followed by 40 cycles of 15 seconds at 95°C, 60 seconds at 60°C. Detection was achieved with the StepOne PLUS instrument and software (Applied Biosystems) using the MasterMix PCR Power SYBR Green (Applied Biosystems, catalog 4309155). Each assay was performed in duplicate in the same run. For construction of standard curves, 5-fold dilution series from *E*. *coli* genomic DNA preparations (MilliporeSigma, catalog D4889) were applied to the PCR.

### Metabolomics.

For extraction of aqueous metabolites in serum and milk, 5 μL of serum and 3 μL of milk were mixed with 150 μL of 40:40:20 methanol/acetonitrile/water mixture. Following vortexing and centrifugation at 16,000*g* for 10 minutes at 4°C, 70 μL of the 150 μL extract was loaded to individual vials. Serum and milk extracts were analyzed by quadrupole-orbitrap mass spectrometer (Q Exactive Plus Hybrid, Thermo Fisher Scientific) operating in negative and positive ion mode coupled to hydrophilic interaction chromatography via electrospray ionization to scan from *m/z* 70 to 830 and 140,000 resolution. Liquid chromatography separation was on an Xbridge BEH Amide column (2.1 mm × 150 mm, 2.5 μm particle size, 130 Å pore size; Waters) at 25°C using a gradient of solvent A (5% acetonitrile in water with 20 mM ammonium acetate and 20 mM ammonium hydroxide) and solvent B (100% acetonitrile). The flow rate was 350 μL/min. The liquid chromatography gradient was 0 minute, 75% B; 3 minutes, 75% B; 4 minutes, 50% B; 5 minutes, 10% B; 7 minutes, 10% B; 7.5 minutes, 75% B; 11 minutes, 75% B. The injection volume of the sample was 3 μL. Data analysis was performed with Compound Discover software (ver.3.2, Thermo Fisher Scientific), and data were corrected for multiple comparisons, based on the number of measured metabolites, via the Benjamini-Hochberg method, with an FDR cutoff at 0.05 used to determine statistical significance. Heatmap construction was performed using Multiple Experiment Viewer from The Institute of Genomic Research.

### Cell viability assay.

The embryonic mouse hypothalamic cell line mHypoE-N43/5 (Tebubio, catalog CLU127-A) (60) was cultured in DMEM (MilliporeSigma, D5796) supplemented with 10% fetal bovine serum, 100 U/mL penicillin, and 100 μg/mL streptomycin at 37°C in 5% CO_2_ and a humidified atmosphere. PAG (MilliporeSigma) was diluted in sterile saline. Cells were cultured in 96-well, black, clear-bottom, cell culture–treated plates (Thermo Fisher Scientific) and exposed to PAG (15–400 μM) or saline for 48 hours. Cell viability was determined by measuring live cells using the Cell Counting Kit-8 (Dojindo Molecular Technologies Inc.) per the manufacturer’s directions. The working reagent was allowed to incubate for 2 hours before the plate was read.

### Isolated ARH explant culture and image analysis.

Brains were collected from P4 C57BL/6J WT mice (The Jackson Laboratory) mice and sectioned at a 200 μm thickness with a vibroslicer (Electron Microscopy Sciences) as previously described (24). The ARH was then carefully dissected out of each section under a stereomicroscope. Explants were cultured onto a rat tail collagen matrix (BD Biosciences), and each explant was incubated with fresh modified Basal Medium Eagle (Invitrogen) containing either vehicle (saline) or 400 μM of PAG. After 48 hours, the explants were fixed in paraformaldehyde, and neurites extending from the explants were stained with a mouse anti–β-III tubulin antibody (1:5,000, Covance catalog MMS-435P) as previously described (24).

Images were then acquired using a ZEISS LSM 710 confocal system. Sections of 5 regions of interest (100 × 100 μm) spaced at 100, 200, 300, 400, and 500 μm extending radially from the edge of the ARH explants were acquired with a 10× objective. The image analysis was performed using ImageJ analysis software, as previously described (24). Briefly, each image plane was binarized to isolate labeled fibers from the background and compensate for differences in fluorescence intensity. The integrated intensity, which reflects the total number of pixels in the binarized image, was then calculated for each image. This procedure was conducted for each image plane in the stack, and the values for all of the image planes in a stack were summed.

### Statistics.

All values are represented as the mean ± SEM. Statistical analyses were conducted using GraphPad Prism (version 7.0a). Data sets with only 2 independent groups were analyzed for statistical significance using unpaired 2-tailed Student’s *t* test. Data sets with more than 2 groups were analyzed using a 1-way ANOVA followed by Tukey’s multiple-comparison test. For statistical analyses of body weight, GTTs, and RER, we performed 2-way ANOVAs followed by Bonferroni’s multiple-comparison test. Statistically significant outliers were calculated using Grubbs’s test for outliers. *P* ≤ 0.05 was considered statistically significant.

For the microbiome analysis, 1-way ANOVA was used to compare total bacteria and Shannon’s α-diversity index between the different groups, followed by Tukey’s post hoc test. The β-diversity was represented by multidimensional scaling using weighted UniFrac distance calculations on the rarefied count matrix (R package phyloseq). The data were normalized by rarefaction before diversity analysis. Distances were calculated using the package phyloseq/distance, followed by phyloseq/ordinate and phyloseq/plot_ordination. Statistical analysis was done using permanova/adonis, followed by a pairwise post hoc test. To compare the relative abundance of major bacterial phyla or families, nonparametric ANOVA (Kruskal-Wallis) was followed by Dunn’s post hoc test to show the impact of the treatment on the relative abundance of each phylum/family.

### Study approval.

All animal procedures were conducted in compliance with and approved by the IACUC of The Saban Research Institute of the Children’s Hospital of Los Angeles (Protocol number 404-18) and by the Institutional Ethics Committees of Care and Use of Experimental Animals of the University of Lille 2 (Lille, France). All experiments were performed in accordance with the guidelines for animal use specified by the European Union Council Directive of September 22, 2010 (2010/63/EU), and the approved protocol (APAFIS 13387–2017122712209790 v9) by the Ethical Committee of the French Ministry of Education and Research (Paris, France).

## Author contributions

SP and SGB conceived and designed the project. SP, AMB, MR, MM, AJ, PG, PYB, HB, AK, and SS performed experiments. SGB, SP, AMB, MR, MM, PG, CJ, and AF analyzed data. JP performed the literature review. MIG provided critical expertise. SP, MR, and SGB wrote the manuscript. All the authors read and approved the manuscript.

## Supplementary Material

Supplemental data

## Version 1 04/04/2023

In-Press Preview

## Version 2 05/22/2023

Electronic publication
