# Supplementary material for: Maternal low-calorie sweetener consumption rewires hypothalamic melanocortin circuits via a gut microbial co-metabolite pathway
Source: JCI Insight. 2023 May 22;8(10):e156397. doi: 10.1172/jci.insight.156397 (PMC10322686; doi:10.1172/jci.insight.156397)
Supplement: Supplemental data [file jciinsight-8-156397-s020.pdf]

Supplemental Material

Supplemental Table 1. Existing human studies on the association between maternal LCS intake during pregnancy and offspring body mass index and/or adiposity[A]

**Authors of study**	Azad *et al.*	Zhu *et al.*	Gillman *et al.*	Azad *et al.*	Laforest-Lapointe *et al.*	Plows *et al.*
**Year published**	2016	2017	2017	2020	2021	2022
**Cohort**	CHILD (Canada)	DNBC (Denmark)	Project Viva (United States)	CHILD (Canada)	CHILD (Canada)	Project Viva (United States)
**Study type**	Observational prospective cohort	Observational prospective cohort	Observational prospective cohort	Observational prospective cohort	Observational prospective cohort	Observational prospective cohort
**Years**	2008-2012	1996-2002	1999-2002	2008-2012	2008-2012	1999-2002
**Primary aim of cohort**	"to advance knowledge about the genetic and environmental determinants of atopic diseases including asthma, allergy, allergic rhinitis, and eczema through trans-disciplinary and longitudinal study, with the goal of advancing the health status and well-being of children in Canada"	"to investigate the causal link between exposures in early life and disease later on and the possibilities for disease prevention."	"to examine prenatal diet and other factors in relation to maternal and child health"	"to advance knowledge about the genetic and environmental determinants of atopic diseases including asthma, allergy, allergic rhinitis, and eczema through trans-disciplinary and longitudinal study, with the goal of advancing the health status and well-being of children in Canada"	"to advance knowledge about the genetic and environmental determinants of atopic diseases including asthma, allergy, allergic rhinitis, and eczema through trans-disciplinary and longitudinal study, with the goal of advancing the health status and well-being of children in Canada"	"to examine prenatal diet and other factors in relation to maternal and child health"
**Primary aim of study**	To determine whether maternal consumption of artificially sweetened beverages (ASB) during pregnancy is associated with infant body mass index (BMI)	To examine associations between maternal intake of artificially sweetened beverages and sugar-sweetened beverages with offspring growth through age 7 among high-risk children born to women with gestational diabetes	To examine associations of sugar sweetened beverages (SSBs) and other beverage intake during pregnancy with adiposity in midchildhood	"To extend our previous findings on maternal LCS consumption and infant body composition in the longitudinal CHILD cohort by reassessing this relationship at 3 years of age."	"to determine if maternal consumption of artificially sweetened beverages (ASB) during pregnancy is associated with modifications of infant gut bacterial community composition and function during the first year of life, and whether these alterations are linked with infant body mass index (BMI) at one year of age "	To examine the extent to which LCS intake during pregnancy is associated with offspring body mass index (BMI) z-score and body fat longitudinally from birth to 18 years.
**Inclusion criteria**	- Singleton pregnancy<br>- >35 weeks' gestation<br>- Delivered at 1 of 4 participating sites in Canada	- Singleton pregnancy<br>- Danish-speaking<br>- Diagnosis of gestational diabetes	- Singleton pregnancy<br>- >34 weeks' gestation<br>- Born in 1 of 8 obstetric offices in eastern Massachusetts	- Singleton pregnancy<br>- >35 weeks' gestation<br>- Delivered at 1 of 4 participating sites in Canada	- Singleton pregnancy<br>- >35 weeks' gestation<br>- Delivered at 1 of 4 participating sites in Canada	- Singleton pregnancy<br>- >34 weeks' gestation<br>- Born in 1 of 8 obstetric offices in eastern Massachusetts - Complete dietary data

						- At least 1 research-assistant measured weight and length/height OR ≥3 measures of weight and length/height obtained either in research visits or clinical medical records (spanning birth to age 18 years)
**Exclusion criteria**	- Congenital abnormalities	- Missing outcome data<br>- Implausible daily energy intake<br>- Pre-existing diabetes - Recurrent gestational diabetes	- Previous diagnosis of maternal type 1, 2, or gestational diabetes<br>- Did not attend mid-childhood visit	- Congenital abnormalities	- Congenital abnormalities<br>- Antibiotics before three months old	- Previous diagnosis of maternal type 1, 2, or gestational diabetes
**Participants in analysis**	3033 mother-infant dyads of the 3542 total in the CHILD cohort	918 mother-child pairs of the 101,042 total in the DNBC cohort	1078 mother-child pairs of the 2128 total in the Project Viva cohort	2298 mother-child dyads of the 3542 total in the CHILD cohort	A subset of 100 infants from the 3621 dyads in the CHILD cohort divided equally between mothers that reported little or no ASB consumption (< 1/month) or daily ASB consumption during pregnancy. Groups balanced for infant sex, birth mode, breastfeeding at three and 12 months, maternal BMI, and antibiotic use in infants before 12 months.	1683 mother-child pairs of the 2128 total in the Project Viva cohort
**Race/ethnicity of participants in analysis**	Not reported	Not reported	- 67.7 % White<br>- 16.4% Black<br>- 6.5% Hispanic<br>- 5.0% Asian<br>- 4.5% Other	Not reported	- 74.0 % White non-consumers vs 88.0% White daily consumers<br>- 14.0% Asian non-consumers vs 6.0% Asian daily consumers<br>- 6.0% First Nations non-consumers vs 4.0% First Nations daily consumers<br>6.0% Other non-consumers vs 2.0% Other daily consumers	- 70.0% White<br>- 14.0% Black<br>- 7.0% Hispanic<br>- 5.0% Asian<br>- 4.0% Other
**Exposure**	Self-reported consumption of "diet soft drinks or pop"	Self-reported consumption of sugar-free/light drinks ranging from	Self-reported consumption of "diet soda" endorsed from	Self-reported consumption of "diet soft drinks or pop"	Self-reported consumption of "diet soft drinks or pop" (1	Self-reported consumption of "diet soda" endorsed from

	(1 serving = 12 oz or 1 can) and "artificial sweetener added to tea or coffee" (1 serving = 1 packet) using a food frequency questionnaire (FFQ) administered in the second or third trimester of pregnancy.	never to ≥ 8 servings/day (1 serving = 1 cup/250 mL) via a FFQ at 25 weeks' gestation. A randomly selected sample of 103 women completed another FFQ at 33-35 weeks' gestation.	"never/less than 1 per month" to "4 or more cans per day" in a semi-quantitative FFQ administered in the first and second trimesters of pregnancy.	(1 serving = 12 oz or 1 can) and "artificial sweetener added to tea or coffee" (1 serving = 1 packet) using a food frequency questionnaire (FFQ) administered in the second or third trimester of pregnancy.	serving = 12 oz or 1 can) and "artificial sweetener added to tea or coffee" (1 serving = 1 packet) using a food frequency questionnaire (FFQ) administered in the second or third trimester of pregnancy.	"never/less than 1 per month" to "4 or more cans per day" AND "Nutrasweet/Equal" consumption endorsed from "never/less than 1 per month" to "4 or more packets a day" in a semi-quantitative FFQ administered in the first and second trimesters of pregnancy and averaged.
**Outcome(s)**	- Infant BMI (kg/m2)	- Large-for-gestational-age<br>- BMI z-score<br>- Childhood overweight/obesity	- BMI z score<br>- Fat mass index (FMI, kg/m2) from dual-energy radiograph absorptiometry (DEXA)<br>- Sum of subscapular (SS) and triceps (TR) skinfold thicknesses - Central adiposity (SS:TR ratio and waist circumference).	- BMI z-score<br>- subscapular skinfolds	- Infant gut bacterial community composition during the first year of life<br>- Urine untargeted metabolomics<br>- Infant BMI at one year of age	- BMI z score<br>- BMI trajectory over course of childhood<br>- Fat mass index [FMI, kg/m2] from dual-energy radiograph absorptiometry<br>- Sum of subscapular [SS] and triceps [TR] skinfold thicknesses
**Timepoint**	1 year of age	Birth and 7 years of age	Mid-childhood (median age 7.7 years)	3 years of age	3 and 12 months of age	- Birth<br>- 6 months<br>- 3 years<br>- 7 years<br>- 12 years<br>- 14 years<br>- 18 years
**Covariates**	- Maternal BMI<br>- Maternal healthy eating index score<br>- Maternal total energy intake<br>- Maternal sugar-sweetened beverage (SSB) consumption<br>- Maternal postsecondary education<br>- Maternal smoking and diabetes during pregnancy<br>- Maternal age<br>- Study site<br>- Gestational age<br>- Race/ethnicity<br>- Breastfeeding duration<br>- Infant sex<br>- Introduction	- Maternal age<br>- Highest familial employment level<br>- Pre-pregnancy BMI<br>- Smoking during pregnancy<br>- Maternal physical activity during pregnancy<br>- Maternal total energy intake<br>- Maternal desserts/sweets intake - Maternal oil/margarine/butter intake<br>- Maternal potato intake<br>- Maternal processed meat intake - Maternal refined grains intake<br>- Maternal whole grains intake<br>- Infant sex<br>- Breastfeeding duration<br>- Child SSB consumption	- Maternal pre-pregnancy BMI<br>- Maternal education<br>- Maternal smoking during pregnancy<br>- Maternal age<br>- Maternal race/ethnicity<br>- Parity<br>- Household income<br>- Child age<br>- Child sex	- Maternal total energy intake<br>- Maternal healthy Eating Index score<br>- Maternal sugar-sweetened beverage intake<br>- Maternal postsecondary education<br>- Maternal smoking, and diabetes during pregnancy<br>- Breastfeeding duration<br>- Child sex.<br>- Child screen time<br>- Child fresh and frozen food intake	- Maternal BMI<br>- Maternal breast milk human milk oligosaccharide secretor status<br>- Intrapartum antibiotics<br>- Maternal ethnicity<br>- Gestational diabetes<br>- Maternal age<br>- Maternal education<br>- Birth mode<br>- Breast feeding duration<br>- Breast feeding at 3 months<br>- Infant diet at 3 and 6 months (presence of breastfeeding, formula, and solids)<br>- Number of antibiotic treatments received from 6 to 12 months	- Maternal pre-pregnancy BMI<br>- Maternal education<br>- Maternal smoking during pregnancy<br>- Maternal age<br>- Maternal race/ethnicity<br>- Parity<br>- Paternal education<br>- Paternal BMI

	of solid foods	- Child physical activity			- Infant secretor status<br>- Older siblings<br>- Infant age	
**Prevalence of LCS consumption in cohort**	- 29.5% of women reported LCS consumption in pregnancy.<br>- 5.1% reported daily LCS consumption.	- 45.4% of women reported artificially sweetened beverage consumption during pregnancy<br>- 9.3% reported daily consumption in pregnancy.	- Mean diet soda intake was 0.1 servings/day in the cohort	- 29.9% of women reported LCS consumption in pregnancy.<br>- 5.2% reported daily LCS consumption.	50% (two groups compared – those with no intake and those with daily intake during pregnancy)	20% of participants reported daily LCS intake during pregnancy
**Findings**	- Compared with no consumption, daily maternal LCS consumption was associated with 0.20-unit increase in infant BMI z-score at 1 year of age<br>- Compared with no consumption, daily maternal LCS consumption associated with 2-fold higher risk of infant overweight at 1 year of age.	- Compared with no intake, daily maternal artificially sweetened beverage intake was associated with 1.57 adjusted relative risk of LGA.<br>- Compared with no intake, daily maternal artificially sweetened beverage intake was associated with 0.59-unit increase in BMI z-score at 7 years.<br>- Compared with no intake, daily maternal artificially sweetened beverage intake was associated with 1.93 adjusted relative risk of overweight/obesity at 7 years.<br>- Per-serving-per-day substitution of sugar-sweetened beverages with artificially-sweetened beverages was not associated with a lower risk of overweight/obesity at 7 years.	- Diet soda consumption during pregnancy was not associated with greater BMI z-score or any measure of adiposity in mid-childhood<br>- However, replacing maternal SSB intake with diet soda did not have as great a beneficial effect on mid-childhood BMI z-score than did replacing SSB with water, 100% fruit juice, or milk.	- Compared with no consumption, daily maternal LCS consumption was associated with 0.37-unit increase in infant BMI z-score at 3 years of age	- BMI was higher among ASB-exposed infants<br>- Maternal AB consumption was associated with community-level shifts in the infant gut bacterial taxonomy structure and depletion of several *Bacteroides* sp.<br>- Urine succinate and spermidine levels ar 3 months were higher in ASB-exposed infants.<br>- Succinate mediated 28% of the effect of ASB exposure on BMI at one year of age	- Compared with no intake, daily maternal LCS intake in pregnancy was associated with 0.18-unit higher BMI z-score at infancy, 0.20-unit higher BMI z-score at 3 years, and 0.26-unit higher at 7 years.<br>- The association between maternal LCS intake and infant BMI strengthened with age, and by age 18 there was a 0.58-unit difference in BMI z-score between no intake and daily LCS intake during pregnancy.<br>- Compared with no intake, daily maternal LCS intake in pregnancy was associated with higher sum of skinfolds in early childhood (1.34 mm), mid-childhood (2.56 mm), and early adolescence (2.34 mm).<br>- Compared with no intake, daily maternal LCS intake in pregnancy was associated with a 0.38 kg/m2 higher FMI in mid-childhood
**Sex differences?**	Yes – when analysis was stratified by sex (instead of adjusting for sex), effects of LCS consumption on infant overweight was only apparent in	Yes – when analysis was stratified by sex (instead of adjusting for sex) males but not females offspring were at greater risk for overweight/obesity at 7 years (adjusted relative	No sex differences were observed	No sex differences were observed	Sex differences were not reported	Sex differences were not reported

	male infants (adjusted odds ratio of 3.07 (95% CI (1.41-6.69)) in males vs. 0.45 (0.09-2.33) in females)	risk of 2.31 (1.17-4.58) for males vs 1.64 (0.84-3.21) for females.				

[A]Pubmed search terms were ("non nutritive sweetener*"[Title/Abstract] OR "artificial sweetener*"[Title/Abstract] OR "low calorie sweetener*"[Title/Abstract] OR "alternative sweetener*"[Title/Abstract] OR "diet soda"[Title/Abstract] OR "diet beverage*"[Title/Abstract] OR "sugar-free"[Title/Abstract] OR "sugar free"[Title/Abstract] OR "aspartame"[Title/Abstract] OR "acesulfame-K"[Title/Abstract] OR "acesulfame potassium"[Title/Abstract] OR "stevia"[Title/Abstract] OR "rebaudioside"[Title/Abstract] OR "sucralose"[Title/Abstract]) AND ("pregnan*"[Title/Abstract] OR "gestat*"[Title/Abstract]) AND ("obes*"[Title/Abstract] OR "body mass index"[Title/Abstract] OR "BMI"[All Fields] OR "weight"[Title/Abstract])

Supplemental Figures

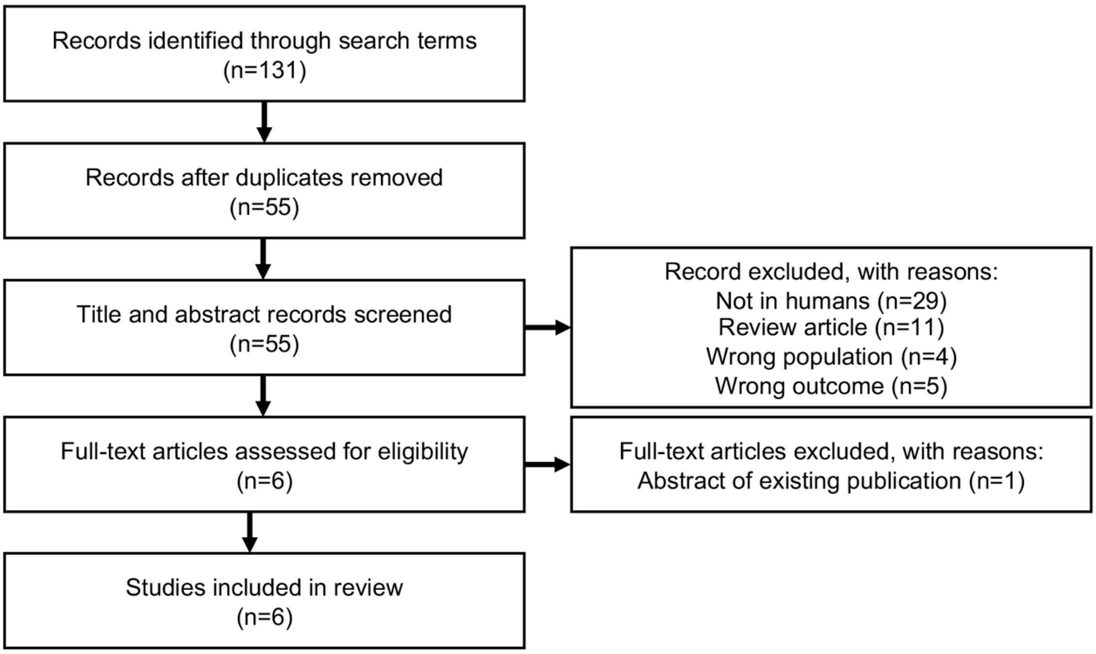

Supplemental Figure 1. Flow diagram for the literature review.

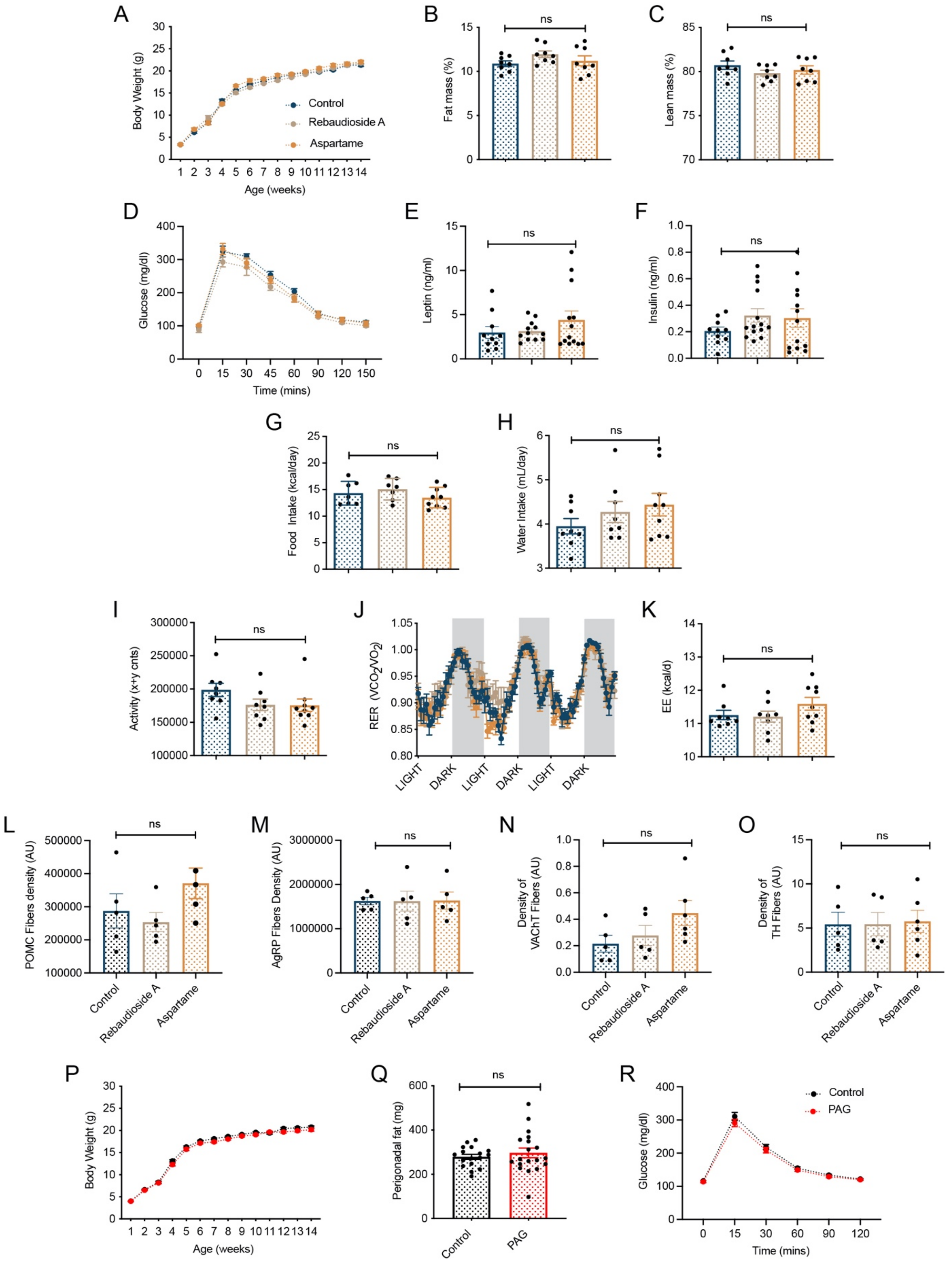

Supplemental Figure 2. Maternal LCS or PAG exposure does not affect female offspring. (A) Body weight curves, (B) fat and (C) lean mass, (D) glucose tolerance tests, and (E) serum leptin and (F) insulin levels of female mice born to dams consuming either a water (control), rebaudioside A, or aspartame solution (*n* = 6-14 animals per group). (G) Food intake, (H) water intake, (I) locomotive activity, (J) respiratory exchange ratio (RER), and (K) energy expenditure in 14-week-old female mice born to dams consuming either a water (control), rebaudioside A,

or aspartame solution (*n* = 6-13 animals per group). (L-O) Quantification of the density of (L) pro-opiomelanocortin (POMC)-, (M) agouti-related peptide (AgRP)-immunoreactive fibers innervating the paraventricular nucleus of the hypothalamus (PVH), and (N) VAChT (vesicular acetylcholine transporter)- and (O) TH (tyrosine hydroxylase)-immunoreactive fibers in insulin$^+$ islets of 14-week-old female mice born to dams consuming either a water (control), rebaudioside A, or aspartame solution (*n* = 4-6 animals per group). (P) Body weight curves, (Q) perigonadal fat mass, and (R) glucose tolerance tests of female mice born to dams treated with phenylacetylglycine (PAG) (*n* = 14-19 animals per group). Data are presented as mean ± sem. Statistical significance between groups was determined by two-way ANOVA followed by Bonferroni's Multiple Comparison test (A, D, J, P, R) or one-way ANOVA followed by Tukey's Multiple Comparison test (B, C, E-I, K-O, Q).

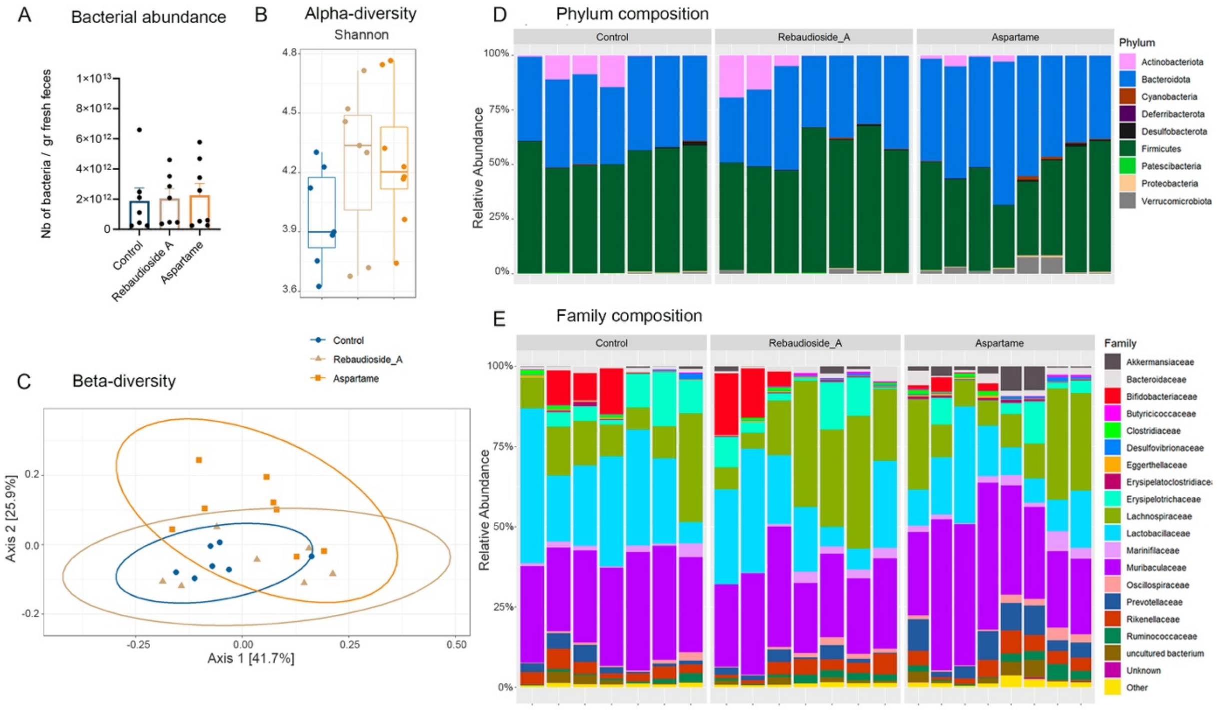

Supplemental Figure 3. Maternal LCS consumption does not affect dams' gut microbiota composition. (A) Abundance of total bacteria, expressed as number of bacteria per gram of fresh feces, in stool of P14 dams consuming either a water (control) (*n* = 7 animals per group), rebaudioside A (*n* = 7 animals per group), or aspartame solution (*n* = 8 animals per group). (B) Boxplot representing the Shannon alpha-diversity index of dams consuming either a water (control) (*n* = 7 animals per group), rebaudioside A (*n* = 7 animals per group), or aspartame solution (*n* = 8 animals per group). (C) Principal coordinates analysis (PCoA) based on wUnifrac index representing the gut microbiota diversity of dams consuming either a water (control) (*n* = 7 animals per group), rebaudioside A (*n* = 7 animals per group), or aspartame solution (*n* = 8 animals per group). Ellipses indicate 95% confidence limits. Relative abundance of (D) major bacteria Phyla and (E) Families in the feces of P14 dams consuming either a water (control) (*n* = 7 animals per group), rebaudioside A (*n* = 7 animals per group), or aspartame solution (*n* = 8 animals per group).

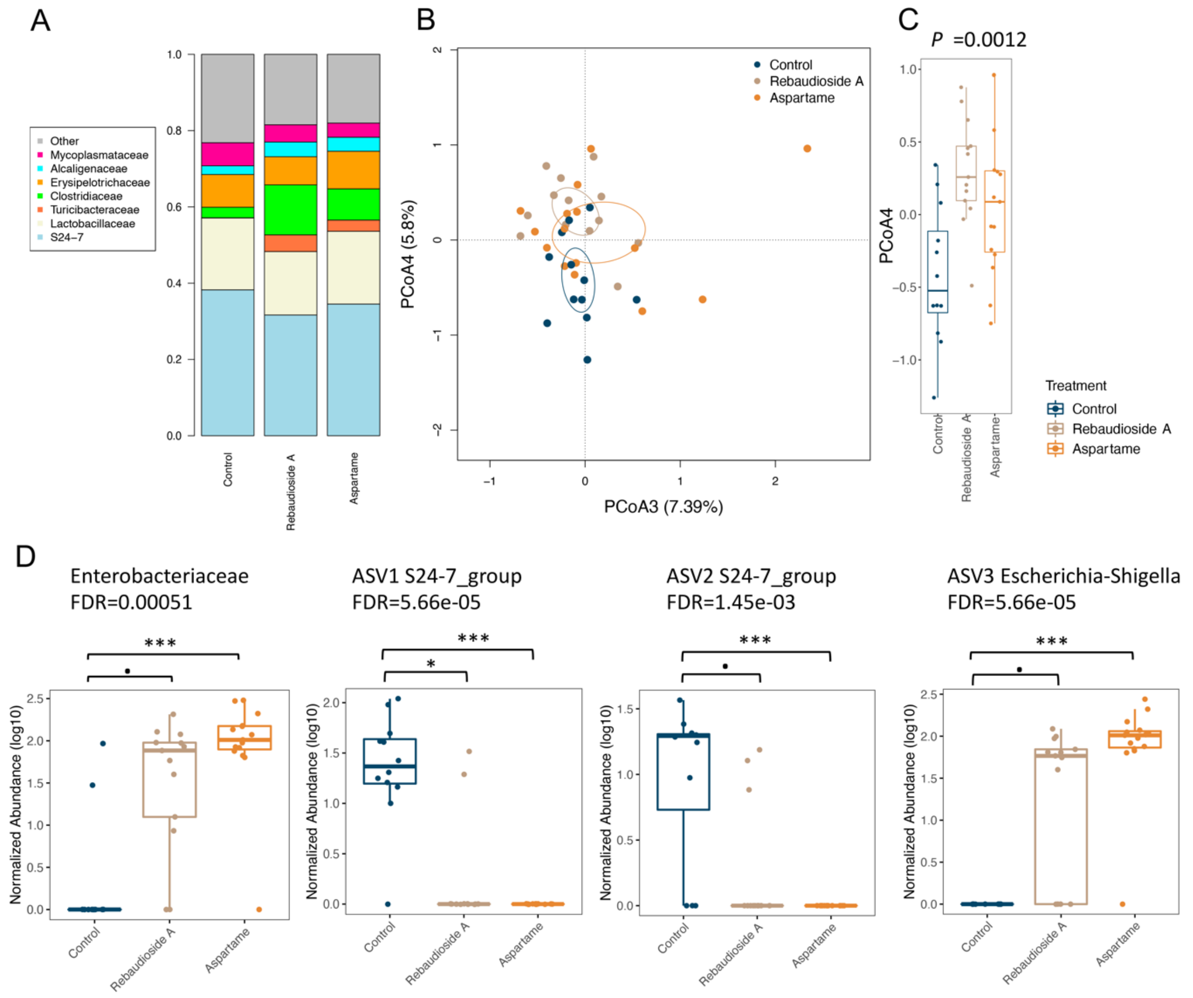

Supplemental Figure 4. Maternal LCS consumption changes the gut microbial composition of offspring mice. (A) Average taxonomic composition of the gut microbiome at the family level of 13- to 14-week-old mice born to dams consuming either a water (control) (*n* = 12 animals per group), rebaudioside A (*n* = 13 animals per group), or aspartame solution (*n* = 15). (B) Principal coordinates analysis (PCoA) based on the Bray-Curtis dissimilarity of species-level gut microbial composition of mice born to dams consuming water (control), rebaudioside A, or aspartame solution. Ellipses indicate 95% confidence limits. (C) The boxplot of sample ordinations at PCoA4, with a significant difference for treatment (ANOVA, P = 0.0012). (D) Log10 normalized abundance of Enterobacteriaceae, two ASVs belonging to order S24-7, and one ASV belonging to *Escherichia-Shigella* in the gut microbiome of mice born to dams consuming either a water (control), rebaudioside A, or aspartame solution. *FDR < 0.1 and ***FDR < 0.001.

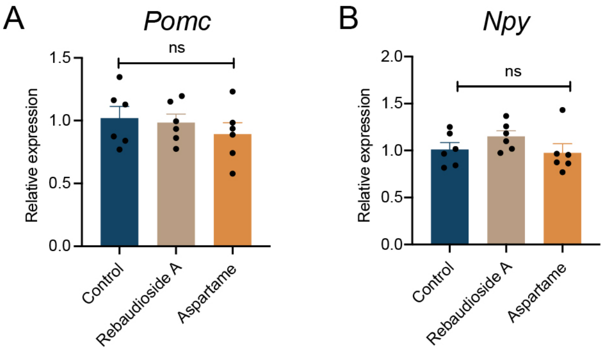

Supplemental Figure 5. Maternal LCS consumption does not affect *Pomc* or *Npy* gene expression. Relative expression of (A) *Pomc* and (B) *Npy* mRNA in the arcuate nucleus of P10 male mice born to dams consuming either a water (control), rebaudioside A, or aspartame solution (n = 6 animals per group). Data are presented as mean + sem. Statistical significance between groups was determined by one-way ANOVA followed by Tukey's Multiple Comparison test.
